# Supplementary material for: Competition for the nascent leading strand shapes the requirements for PCNA loading in the replisome
Source: EMBO J. 2025 Feb 28;44(8):2298–322. doi: 10.1038/s44318-025-00386-4 (PMC12000384; doi:10.1038/s44318-025-00386-4)
Supplement: Supplementary file 1 — Appendix [file 44318_2025_386_MOESM1_ESM.pdf]

Appendix

Competition for the nascent leading strand shapes the requirements for PCNA loading in the replisome

Emma E Fletcher, Morgan L Jones and Joseph TP Yeeles

Table of Contents

Appendix Figure S1	Page 2
Appendix Figure S2	Page 3
Appendix Figure S3	Page 4
Appendix Figure S4	Page 5
Appendix Figure S5	Page 6
Appendix Table S1	Page 7

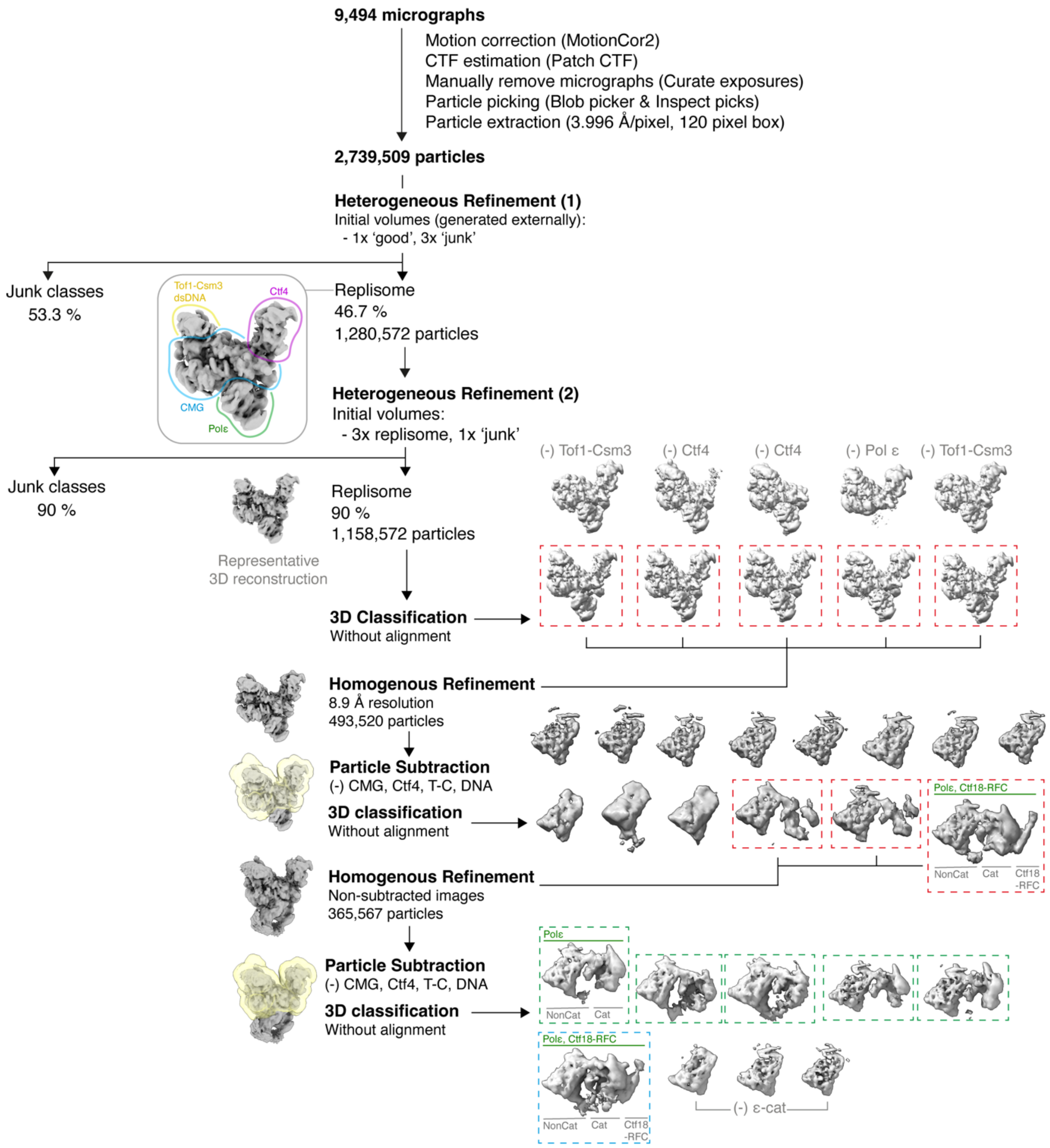

Appendix Figure S1. (Related to Figure 5). Data processing pipeline for cryo-EM reconstructions of the budding yeast replisome prepared with Ctf18-RFC

Data processing is described up to the point where the dataset is split based on the presence or absence of density for Ctf18-Dcc1-Ctf8 bound to the Pol-ε catalytic domain. With each processing step, 3D classes selected for further analysis are enclosed in dashed boxes. Reconstructions containing Ctf18-RFC bound to Pol ε catalytic domain are highlighted with blue boxes, with those lacking Ctf18-RFC density marked in green. The approximate positions of specific proteins are labelled in selected 3D reconstructions to aid interpretation. The protein components absent from specific classes after the initial round of 3D classification without alignment are indicated above their corresponding reconstructions in grey. Abbreviations: Cat: Pol-ε catalytic domain. Noncat: Pol-ε non-catalytic domain.

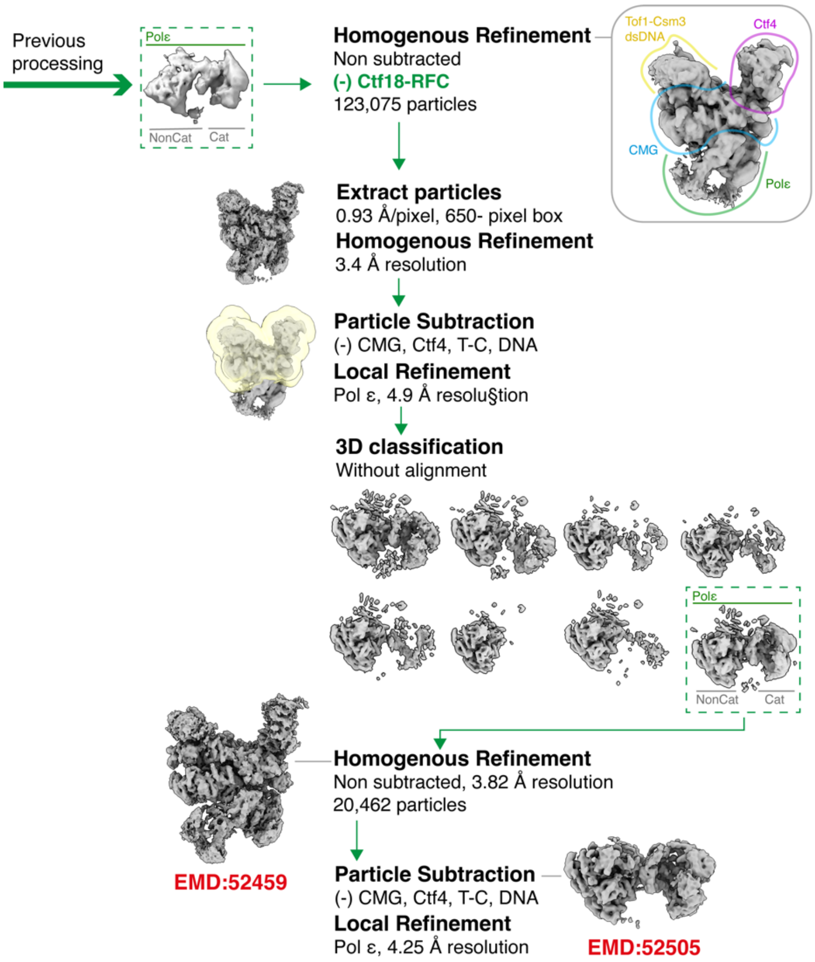

Appendix Figure S2. (Related to Figure 5). Data processing pipeline for the budding yeast replisome, focusing on particles lacking Ctf18-RFC density
Data is displayed and described as in Appendix Figure S1. Unique EMD identifiers for specific maps are included in red bold text.

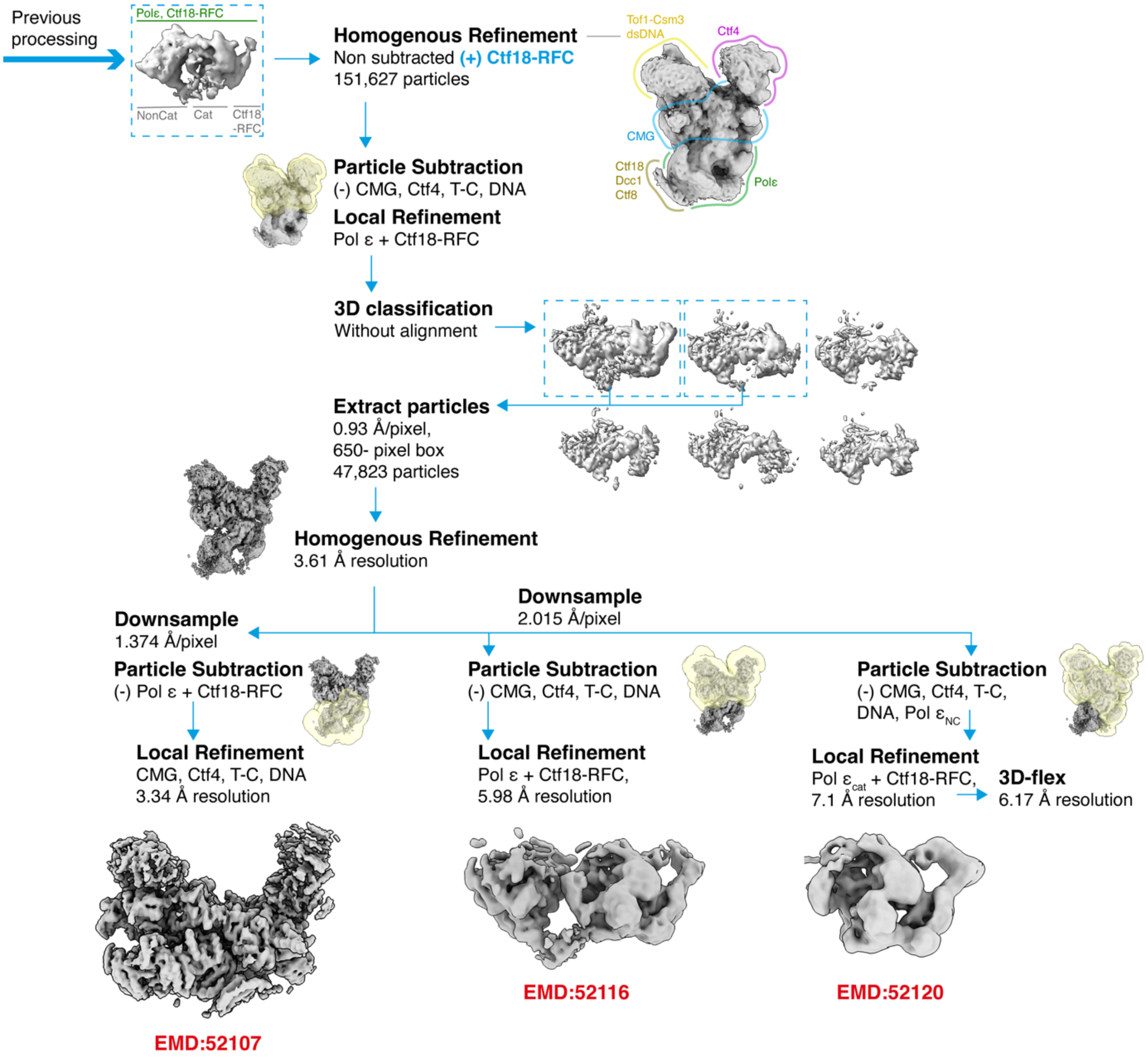

Appendix Figure S3. (Related to Figure 5). Data processing pipeline for the budding yeast replisome, focusing on particles containing Ctf18-RFC density

Data is displayed and described as in Appendix Figure S1. Unique EMD identifiers for specific maps are included in red bold text.

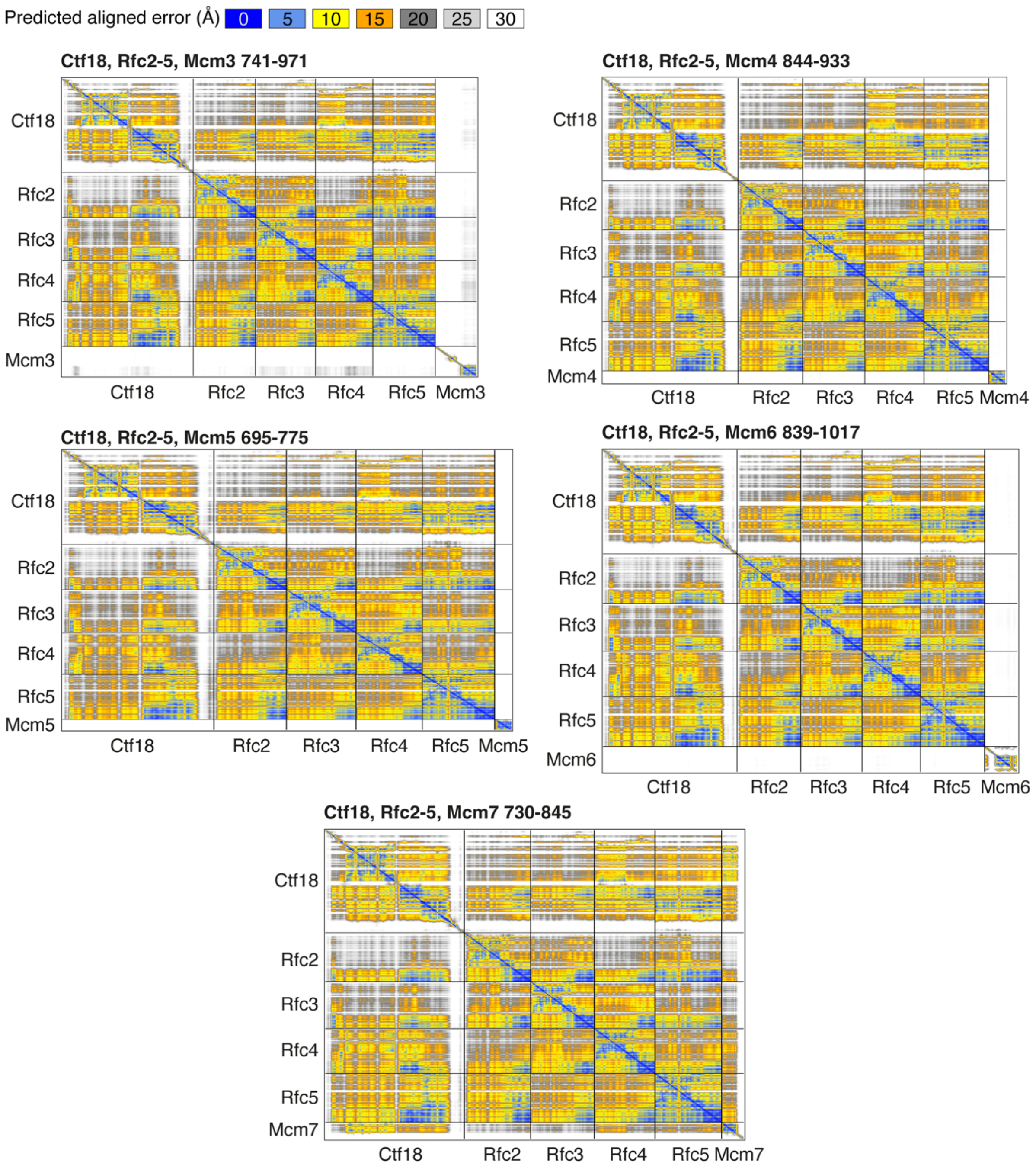

Appendix Figure S4. (Related to Figure 6). Predicted aligned error plots for AlphaFold 3 structure predictions between Ctf18, Rfc2-5 and MCM winged-helices

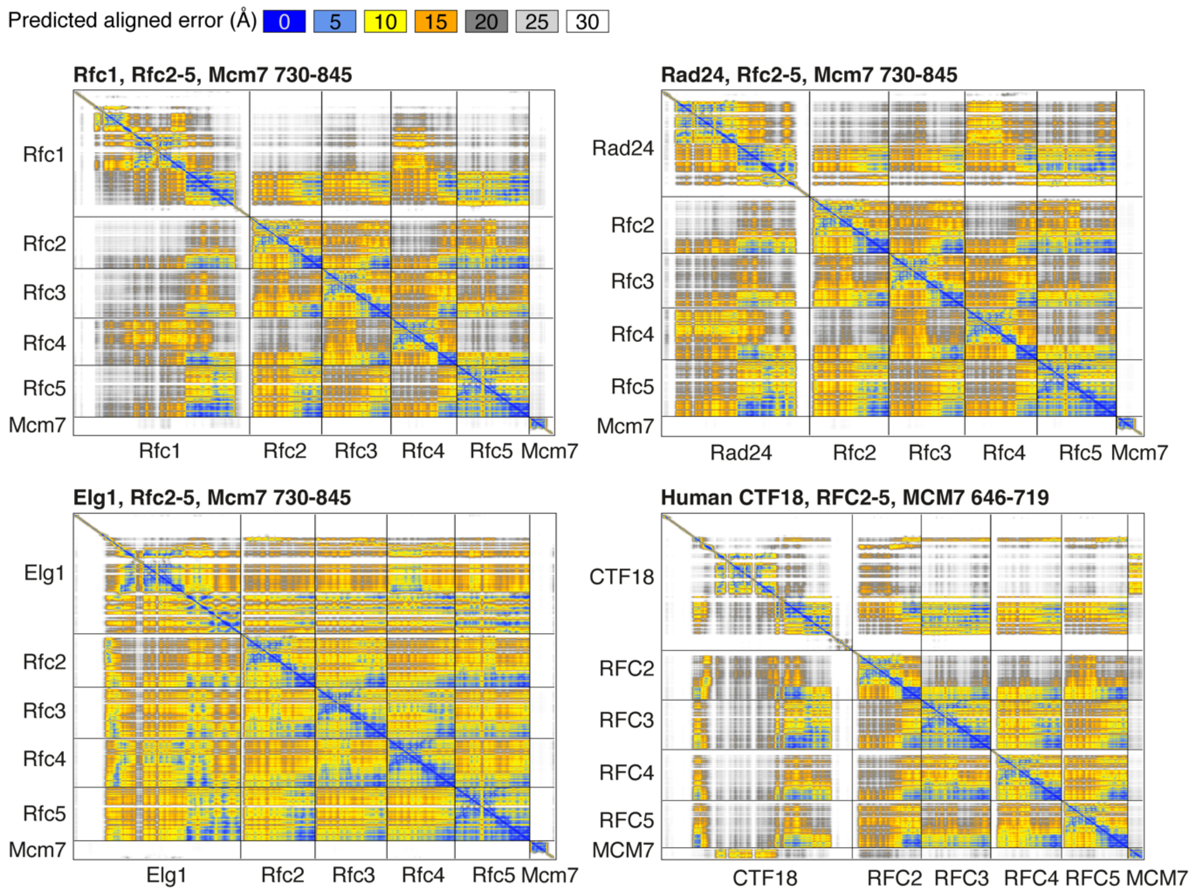

Appendix Figure S5. (Related to Figure 6). Predicted aligned error plots for AlphaFold 3 structure predictions between clamp loaders and the winged-helix of Mcm7

Protein	Affinity Tag	Purified as in	Purification steps
Cdc45	Internal 2xFLAG tag	(Yeeles *et al.*, 2015)	Anti-FLAG M2 Affinity Gel<br>Bio-Gel HT Hydroxyapatite
Cdc6	N-terminal cleavable GST tag	(Coster *et al.*, 2014)	Glutathione Sepharose 4B<br>Bio-Gel HT Hydroxyapatite
Cdt1.Mcm2-7	N-terminal cleavable CBP tag on Mcm3	(Coster *et al.*, 2014)	Calmodulin Sepharose 4B<br>Superdex 200 Increase 10/300 GL
Ctf4	N-terminal CBP tag	(Yeeles *et al.*, 2015)	Calmodulin Sepharose 4B<br>MonoQ 5/50 GL<br>Superdex 200 Increase 10/300 GL
DDK	CBP tag on Dbf4	(On *et al*, 2014)	Calmodulin Sepharose 4B<br>Lambda phosphatase dephosphorylation<br>Superdex 200 Increase 10/300 GL
Dpb11	C-terminal 3xFLAG tag	(Yeeles *et al.*, 2015)	Anti-FLAG M2 Affinity Gel<br>MonoS 5/50 GL
GINS	N-terminal His tag on Psf3	(Yeeles *et al.*, 2015)	Ni-NTA Agarose<br>MonoQ 5/50 GL<br>Superdex 200 Increase 10/300 GL
Mcm10	N-terminal 6xHis tag	(Yeeles *et al.*, 2015)	Ni-NTA Agarose<br>MonoS 5/50 GL (twice)
Mrc1	C-terminal 2xFLAG tag	(Yeeles *et al.*, 2017)	Anti-Flag M2 Affinity Gel<br>Superose 6 Increase 10/300 GL
ORC	Cleavable CBP tag on Orc1	(Frigola *et al.*, 2013)	Calmodulin Sepharose 4B<br>Superdex 200 Increase 10/300 GL
PCNA	Untagged	(Yeeles *et al.*, 2017)	Nucleic acid precipitation with Polymin P<br>Ammonium sulphate precipitation<br>HiTrap DEAE Fast Flow<br>MonoQ 5/50 GL (twice)<br>HiTrap Q FF<br>Superdex 200 Increase 10/300 GL
Pol α-primase	N-terminal CBP tag on Pri1	(Yeeles *et al.*, 2017)	Calmodulin Sepharose 4B<br>MonoQ 5/50 GL<br>Superdex 200 Increase 10/300 GL
Pol δ	C-terminal CBP tag on Pol32	(Yeeles *et al.*, 2017)	Calmodulin Sepharose 4B<br>HiTrap Heparin HP<br>Superdex 200 Increase 10/300 GL
Pol $\delta^{CAT\text{-}DEAD}$	C-terminal CBP tag on Pol32	(Aria & Yeeles, 2018)	Nucleic acid precipitation with Polymin P<br>Ammonium sulphate precipitation<br>Calmodulin Sepharose 4B<br>MonoQ 5/50 GL

Pol ε/ Pol $ε^{PIP}$/ Pol $ε^{exo-}$	C-terminal CBP tag on Dpb4 (3xFLAG tag on endogenous Pol ε)	(Yeeles *et al.*, 2015) (Aria & Yeeles, 2018) (Guilliam & Yeeles, 2021)	Calmodulin Sepharose 4B<br>(Anti-FLAG M2 Affinity Gel)<br>HiTrap Heparin HP<br>Superdex 200 Increase 10/300 GL
RFC	N-terminal CBP tag on Rfc3	(Yeeles *et al.*, 2017)	Calmodulin Sepharose 4B<br>MonoS 5/50 GL<br>Superdex 200 Increase 10/300 GL
RPA	Untagged	(Baretic *et al.*, 2020)	HiTrap Blue HP (twice)<br>ssDNA Cellulose<br>MonoQ 5/50 GL
S-CDK (Δ1-100 Clb5)	N-terminal cleavable CBP tag on Clb5	(Hill *et al*, 2020)	Calmodulin Sepharose 4B<br>Elution by TEV cleavage<br>Superdex 200 Increase 10/300 GL
Sld2	C-terminal 3xFLAG tag	(Yeeles *et al.*, 2015)	Ammonium sulphate precipitation<br>Anti-FLAG M2 Affinity Gel<br>HiTrap SP HP
Sld3/7	C-terminal cleavable TCP tag	(Yeeles *et al.*, 2015)	IgG Sepharose Fast Flow<br>TEV removal with Ni-NTA Agarose<br>Superdex 200 Increase 10/300 GL
Tof1-Csm3	N-terminal cleavable CBP tag on Csm3	(Yeeles *et al.*, 2017)	Calmodulin Sepharose 4B<br>MonoQ 5/50 GL<br>Superdex 200 Increase 10/300 GL

Appendix Table S1. Purification strategies for proteins used in this study
