# Supplementary figures and images for: Competition for the nascent leading strand shapes the requirements for PCNA loading in the replisome
Source: EMBO J. 2025 Feb 28;44(8):2298–322. doi: 10.1038/s44318-025-00386-4 (PMC12000384; doi:10.1038/s44318-025-00386-4)

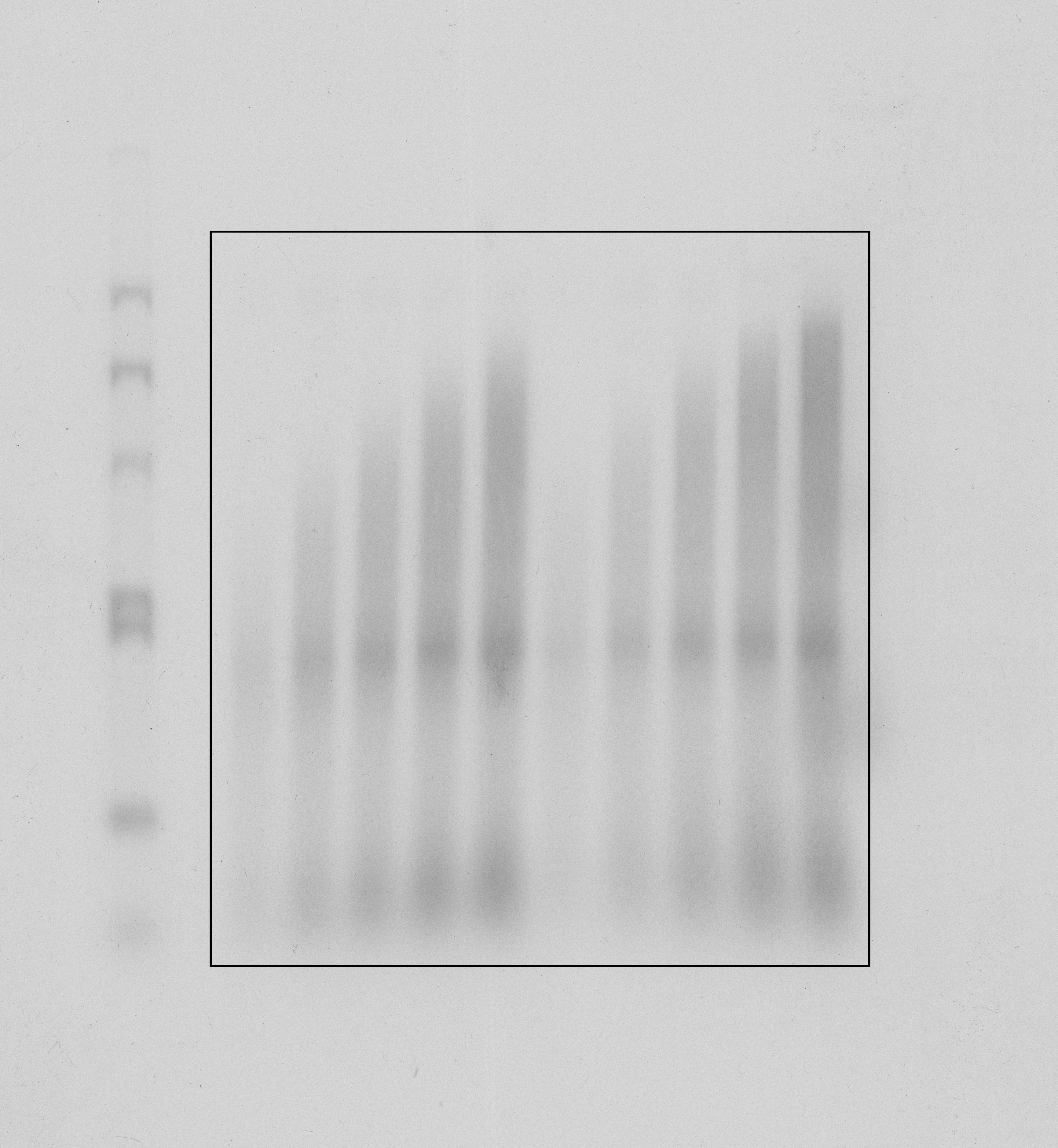

Supplement: Supplementary file 3 — Source data Fig. 1 [file 44318_2025_386_MOESM3_ESM.zip › Figure 1/1B/1B.tif]

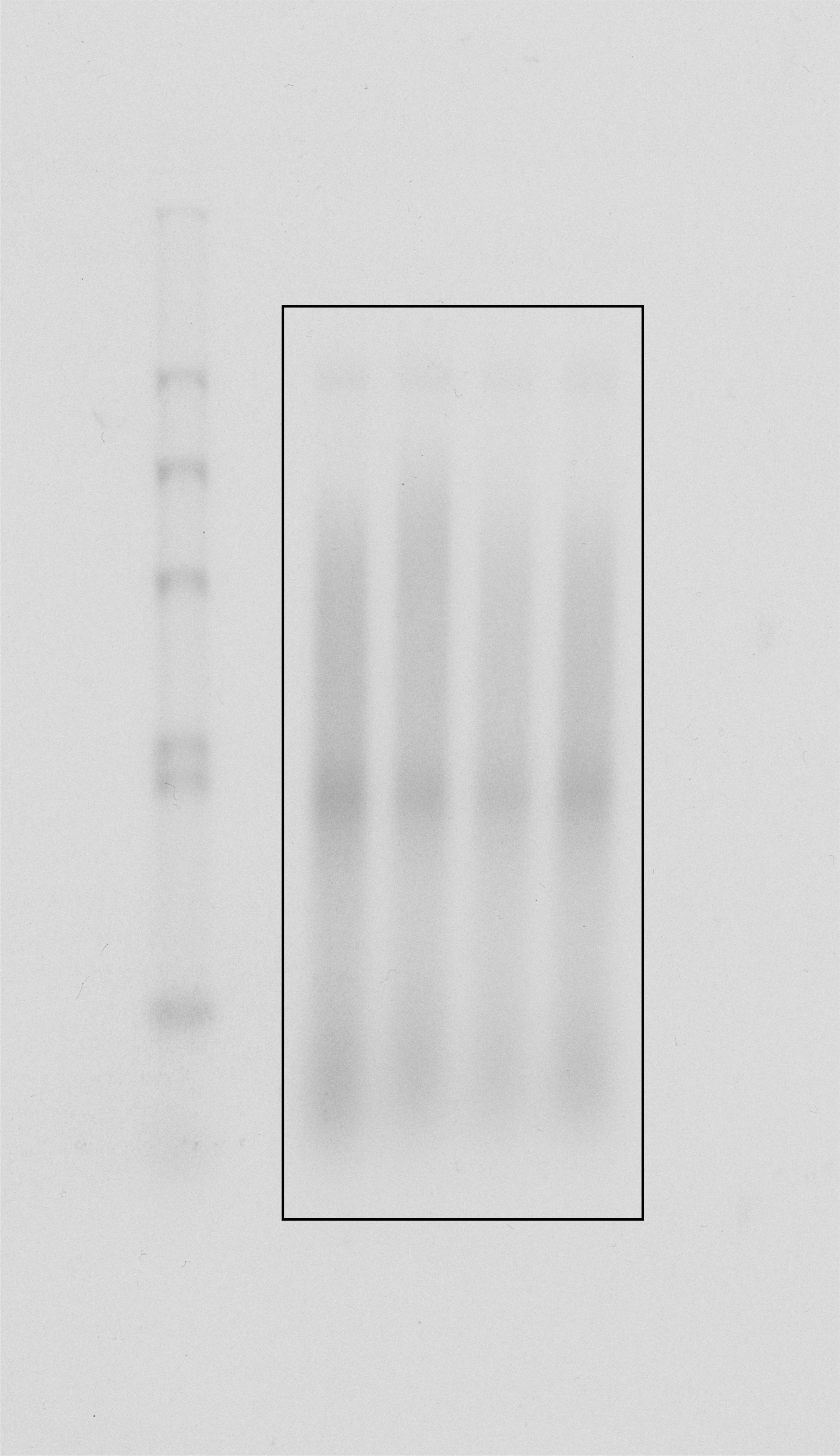

Supplement: Supplementary file 3 — Source data Fig. 1 [file 44318_2025_386_MOESM3_ESM.zip › Figure 1/1E/1E.tif]

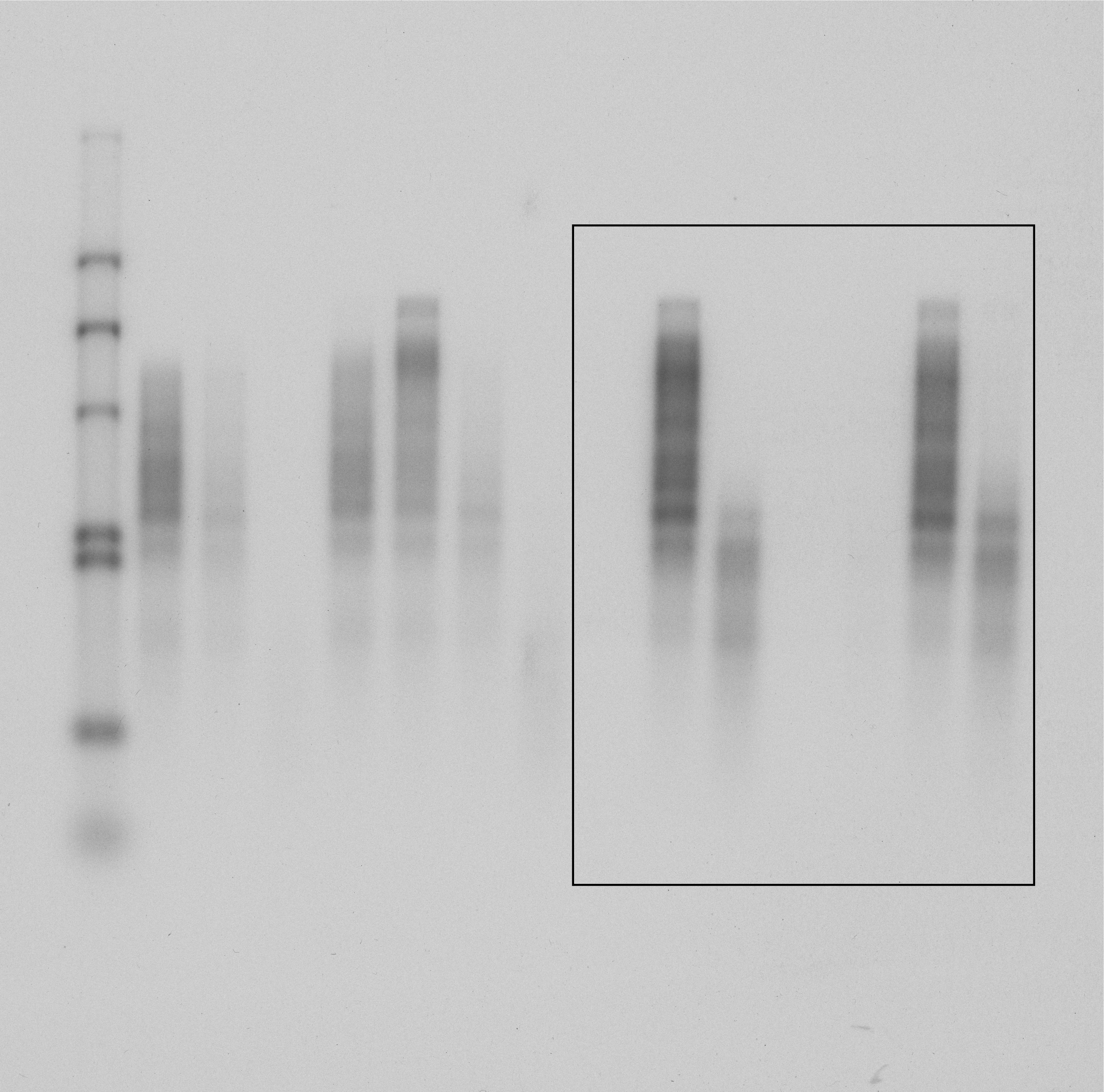

Supplement: Supplementary file 4 — Source data Fig. 2 [file 44318_2025_386_MOESM4_ESM.zip › Figure 2/2C/2C.tif]

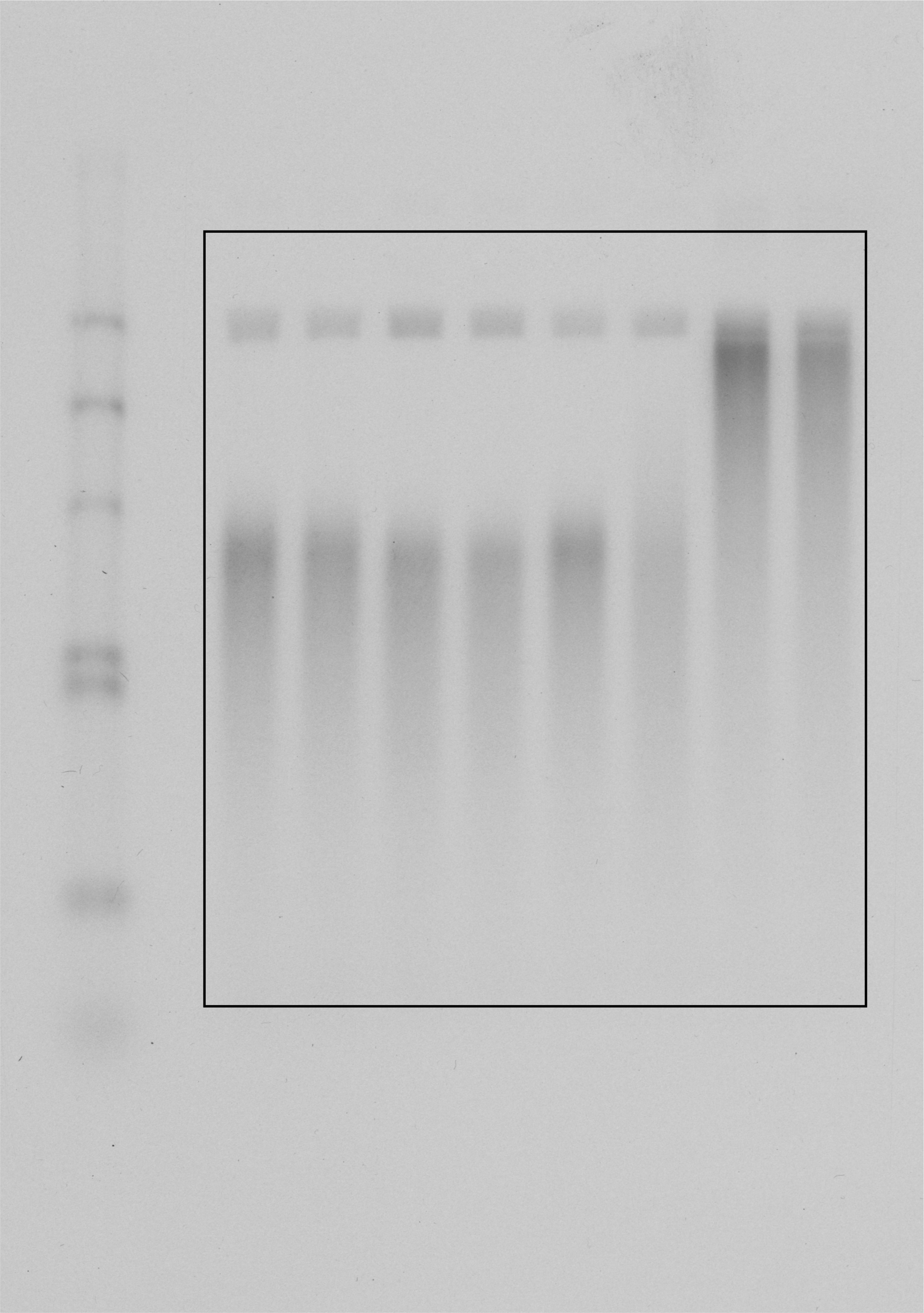

Supplement: Supplementary file 4 — Source data Fig. 2 [file 44318_2025_386_MOESM4_ESM.zip › Figure 2/2B/2B.tif]

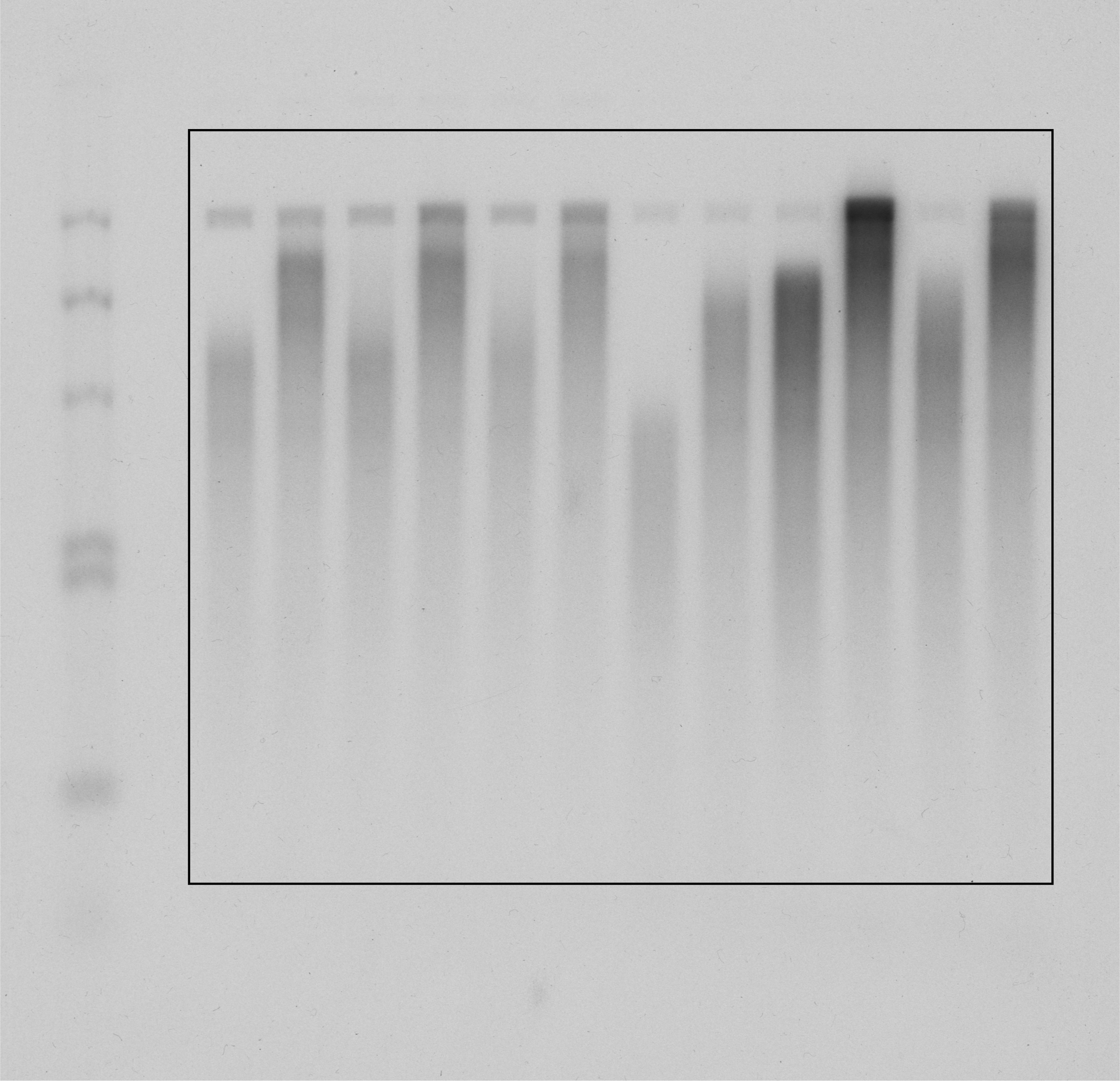

Supplement: Supplementary file 5 — Source data Fig. 3 [file 44318_2025_386_MOESM5_ESM.zip › Figure 3/3C/3C.tif]

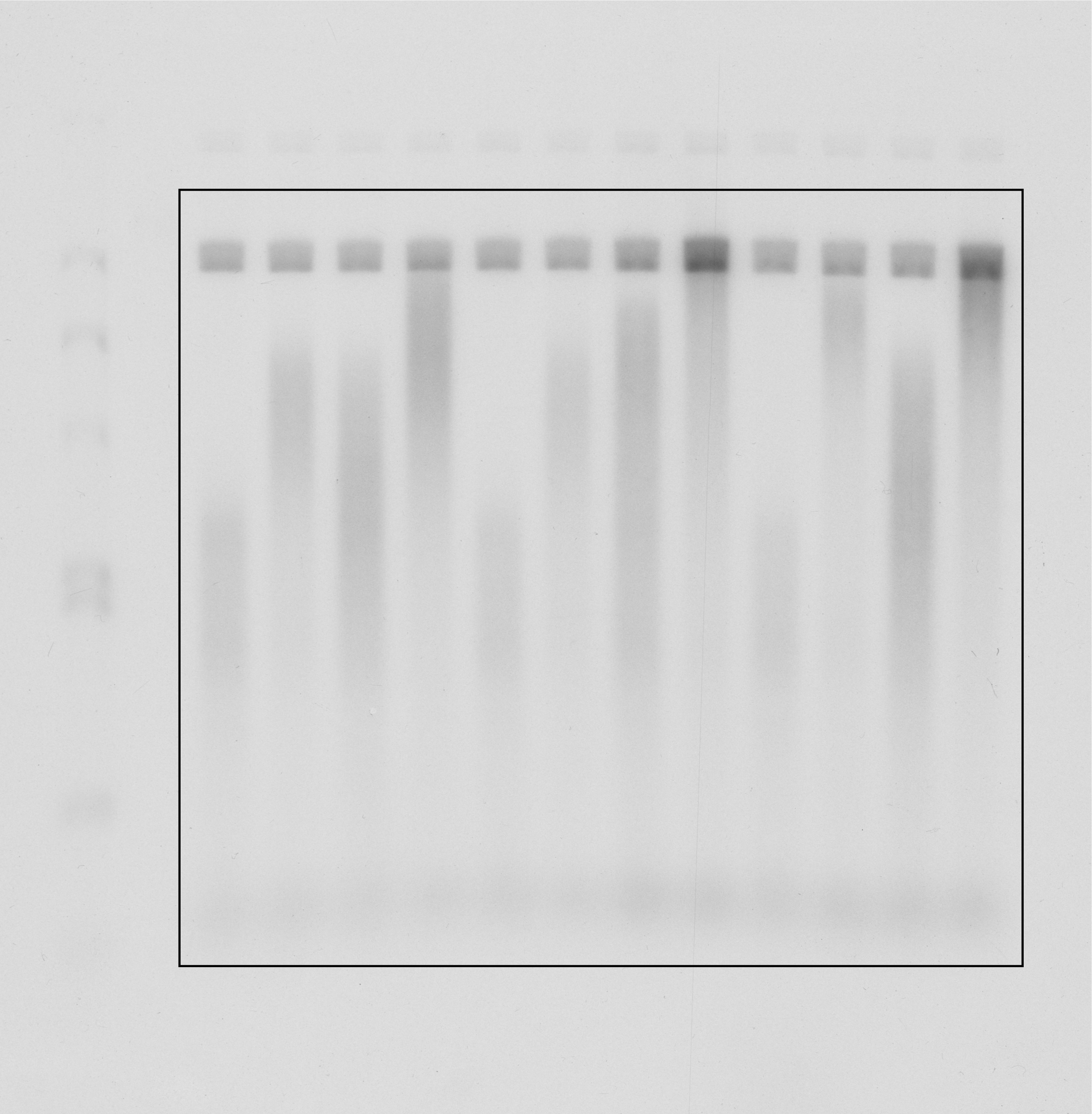

Supplement: Supplementary file 5 — Source data Fig. 3 [file 44318_2025_386_MOESM5_ESM.zip › Figure 3/3A/3A.tif]

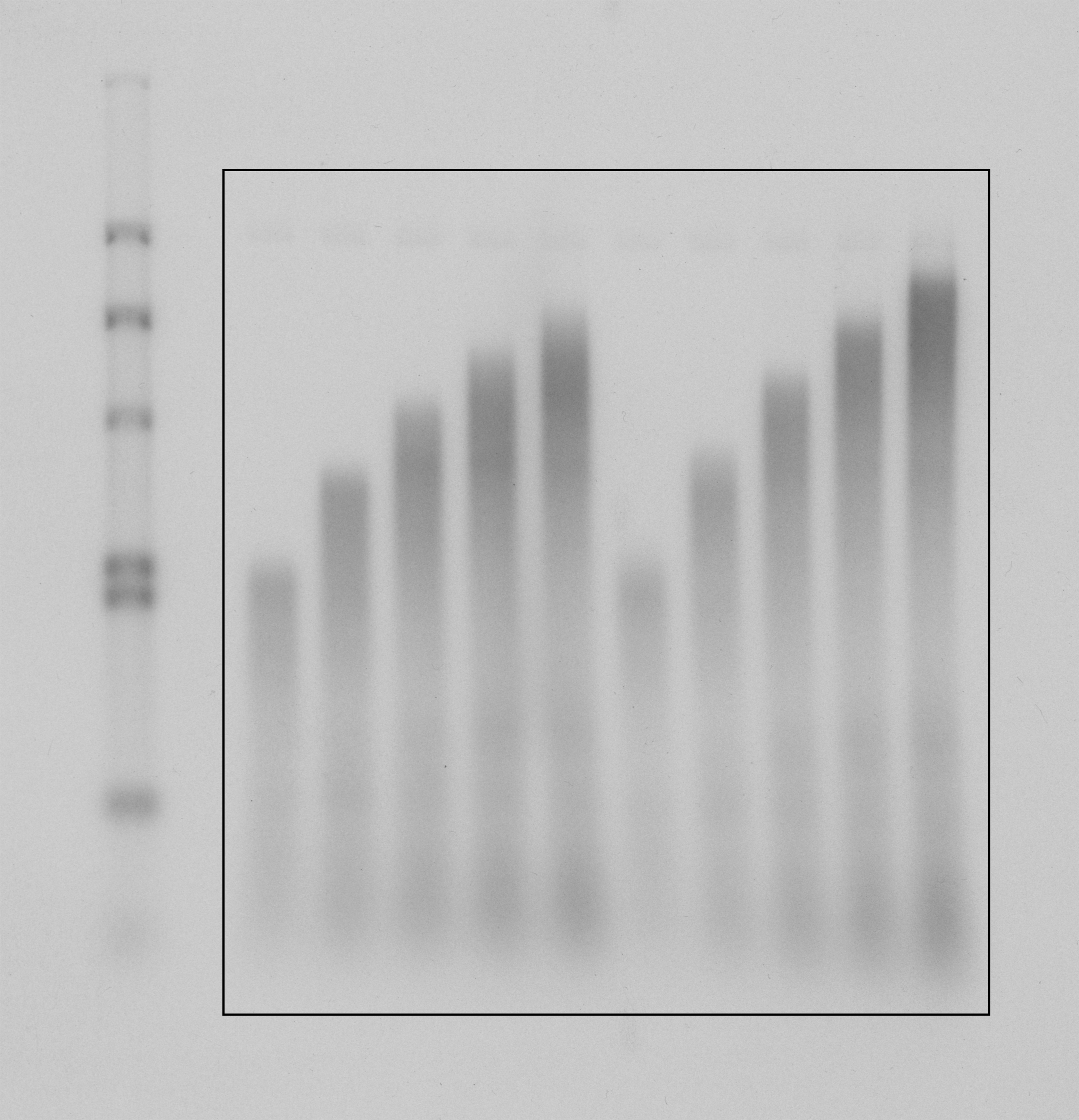

Supplement: Supplementary file 6 — Source data Fig. 4 [file 44318_2025_386_MOESM6_ESM.zip › Figure 4/4B/4B.tif]

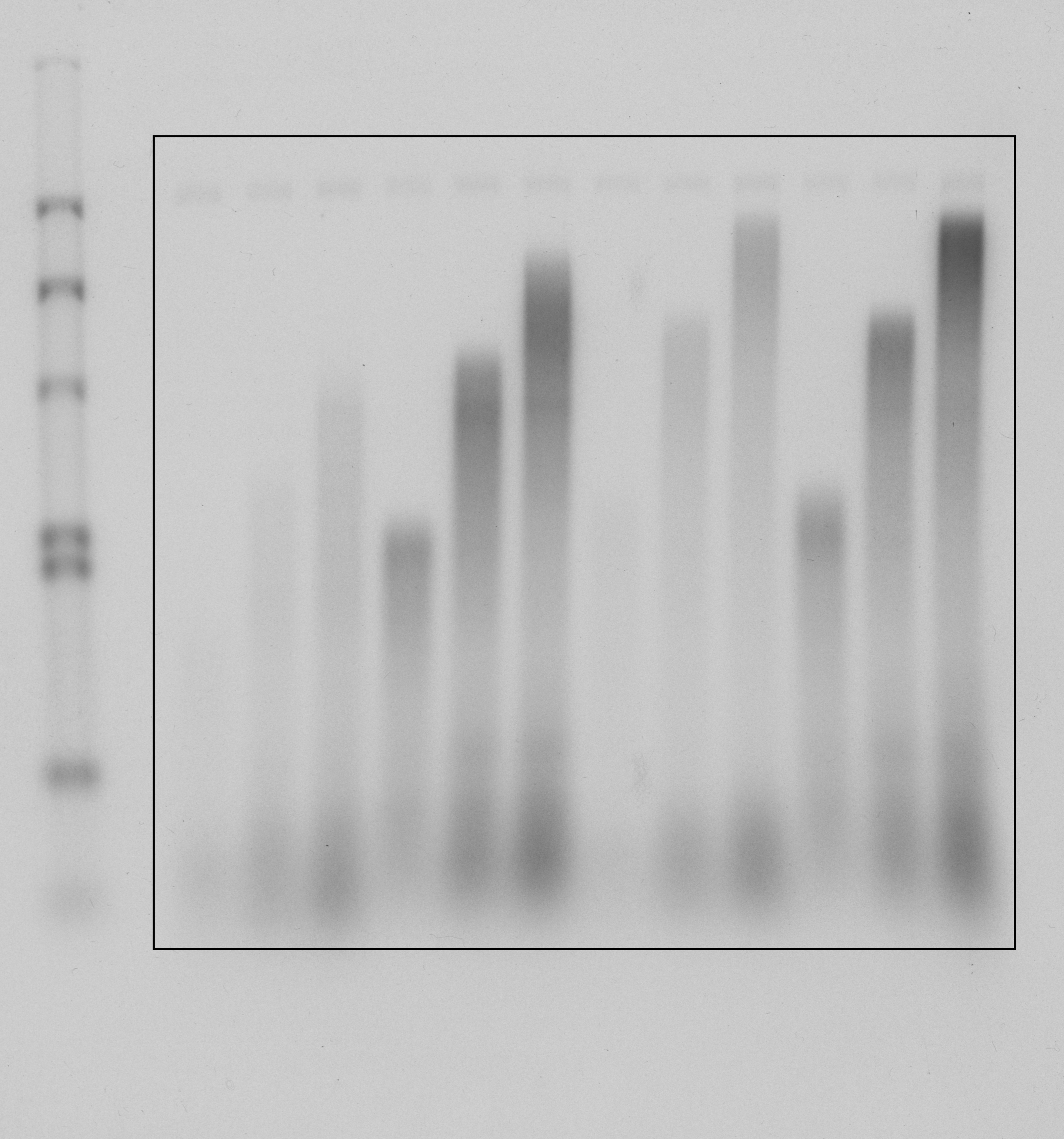

Supplement: Supplementary file 6 — Source data Fig. 4 [file 44318_2025_386_MOESM6_ESM.zip › Figure 4/4D/4D.tif]

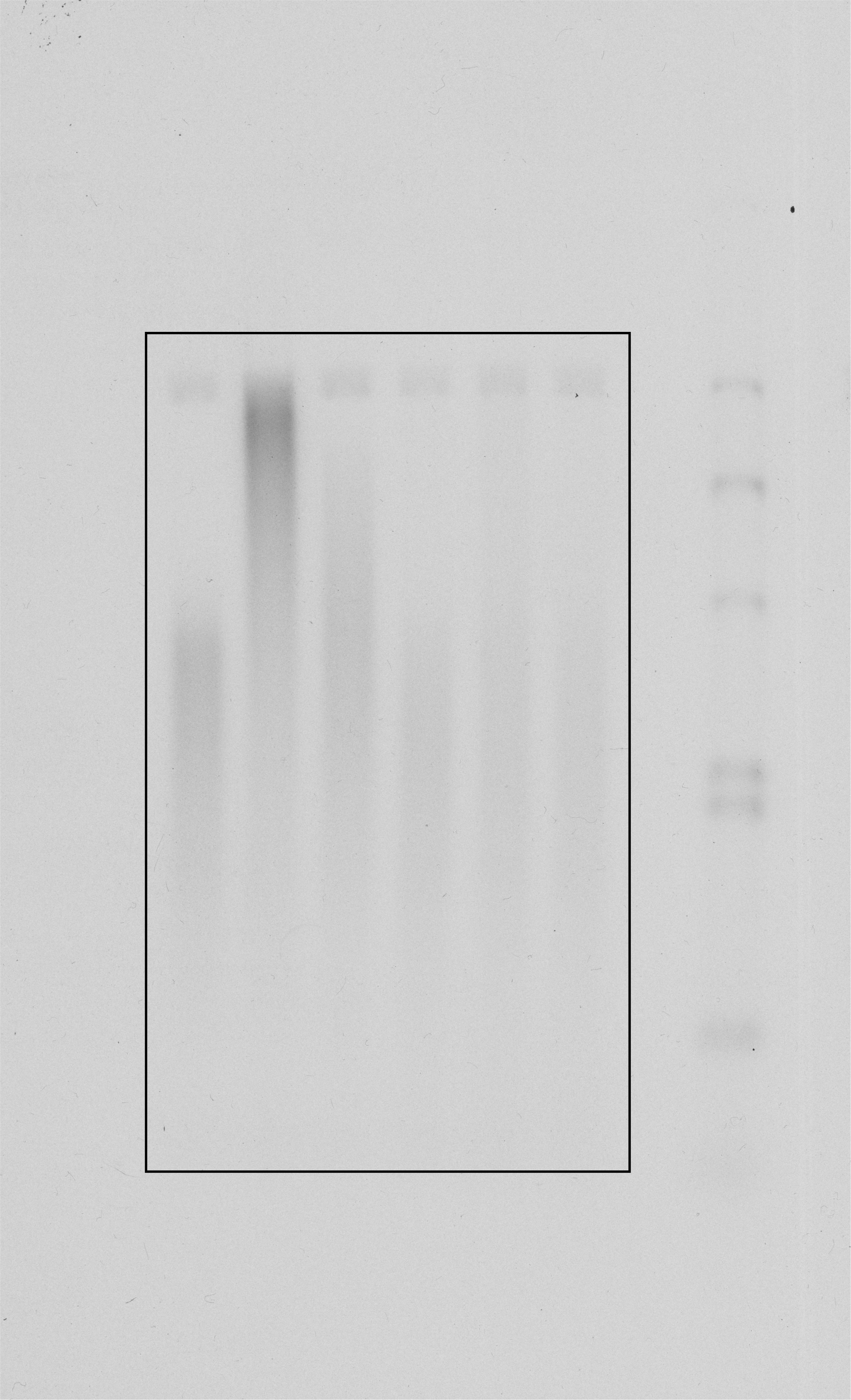

Supplement: Supplementary file 7 — Source data Fig. 6 [file 44318_2025_386_MOESM7_ESM.zip › Figure 6/6C/6C.tif]

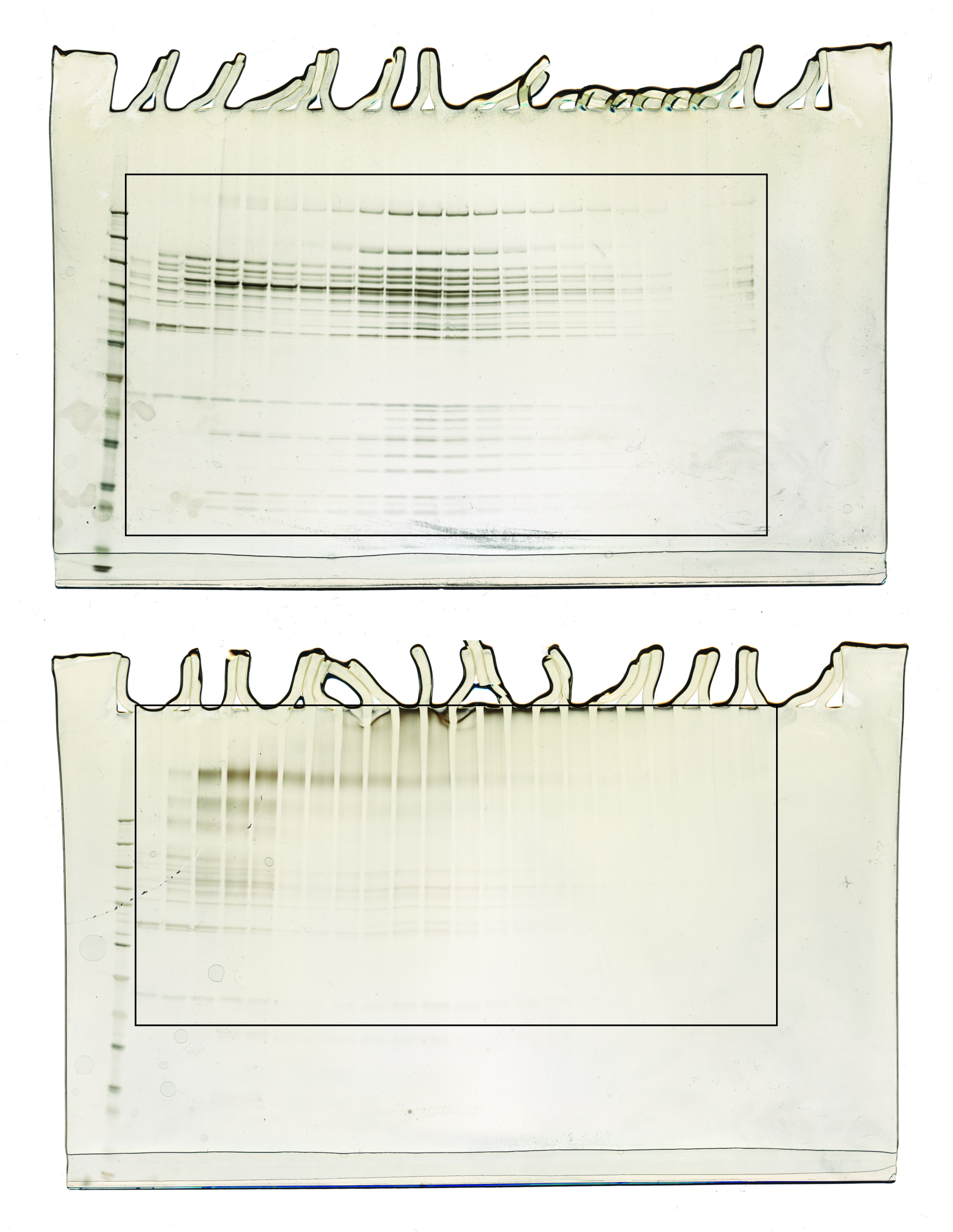

Supplement: Supplementary file 8 — EV Figures Source Data [file 44318_2025_386_MOESM8_ESM.zip › EMBOJ-2024-119352_EVSourceData-2/Figure EV4/EV4A.tif]

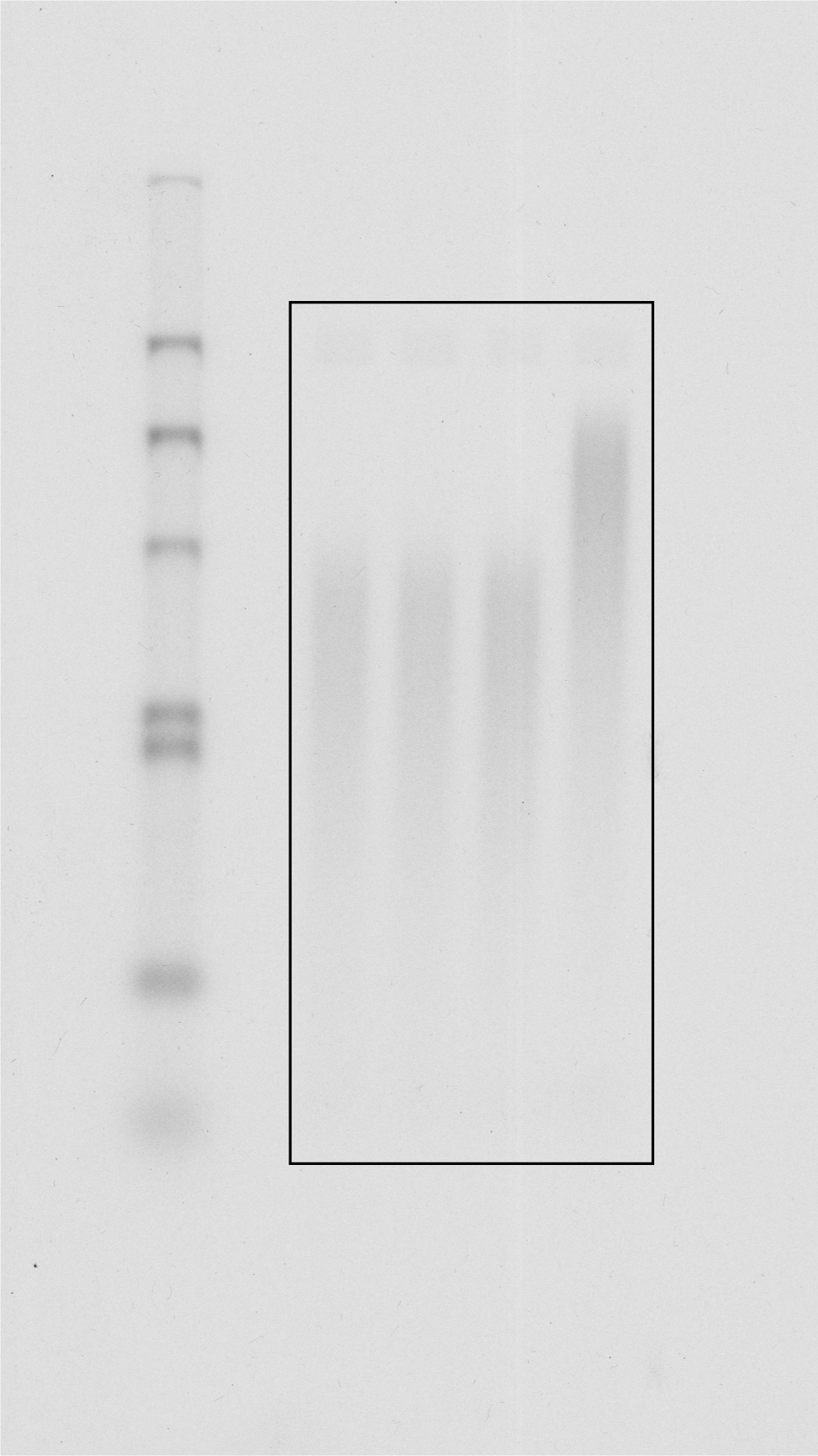

Supplement: Supplementary file 8 — EV Figures Source Data [file 44318_2025_386_MOESM8_ESM.zip › EMBOJ-2024-119352_EVSourceData-2/Figure EV3/EV3B.tif]

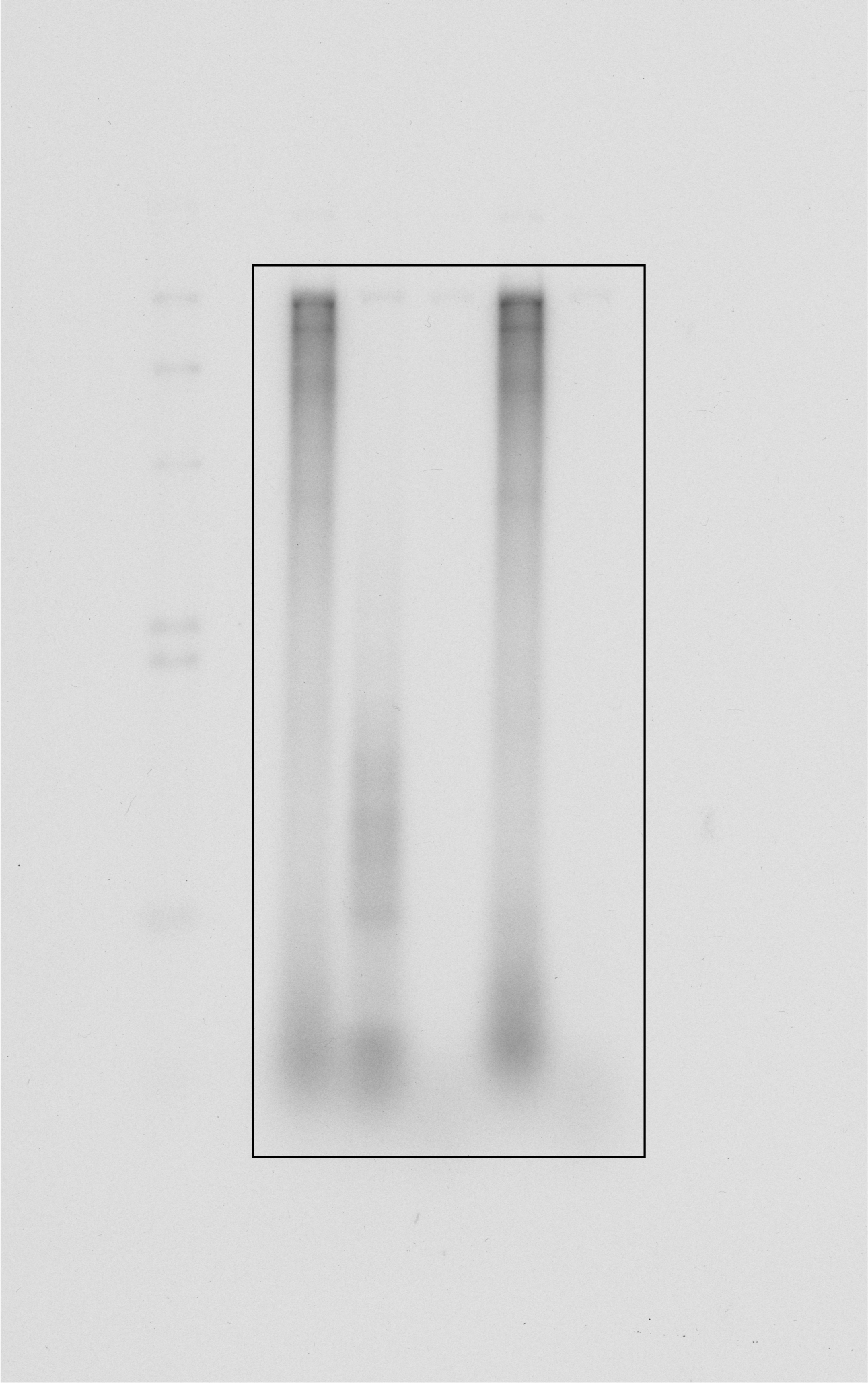

Supplement: Supplementary file 8 — EV Figures Source Data [file 44318_2025_386_MOESM8_ESM.zip › EMBOJ-2024-119352_EVSourceData-2/Figure EV3/EV3C.tif]

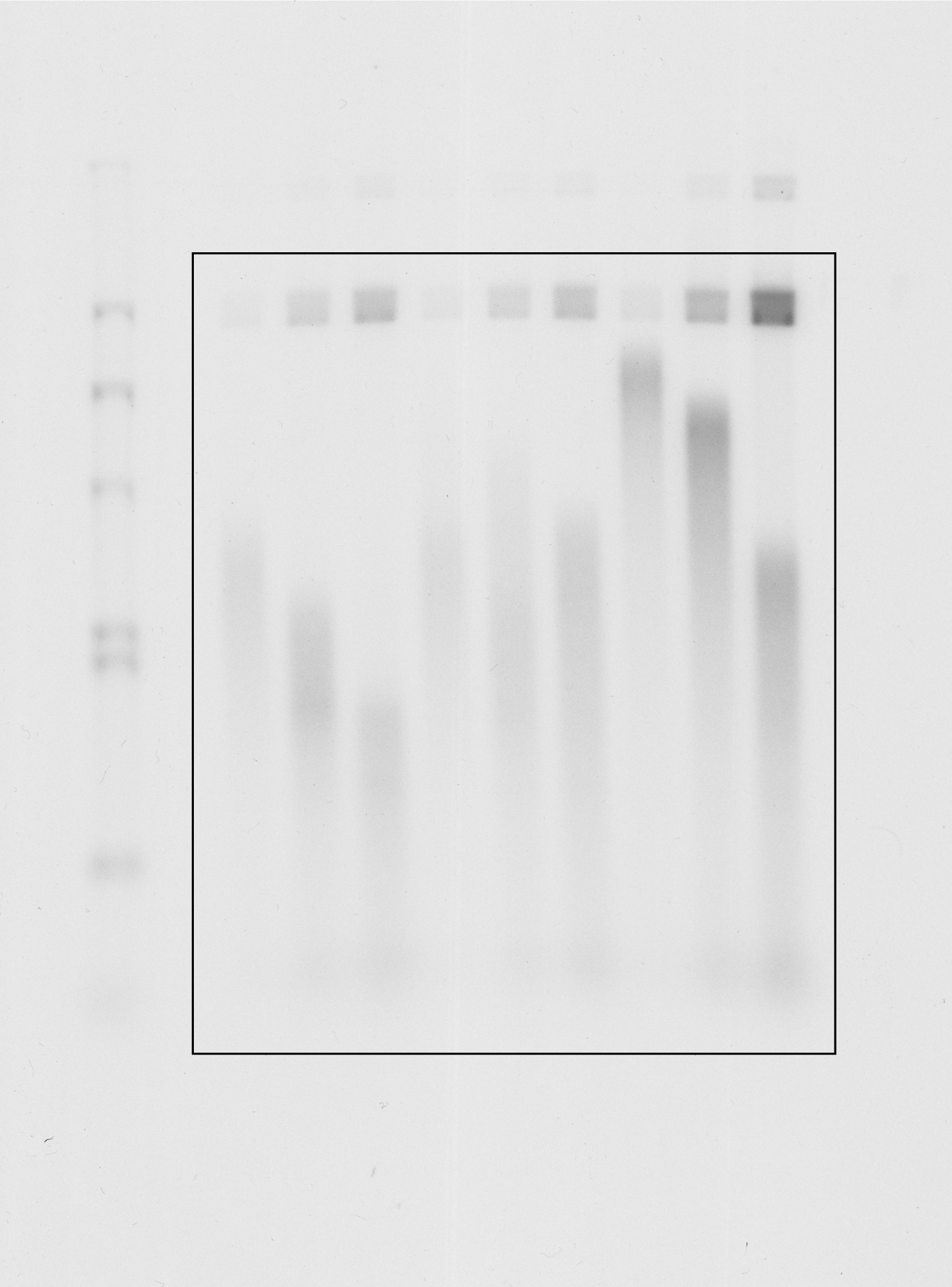

Supplement: Supplementary file 8 — EV Figures Source Data [file 44318_2025_386_MOESM8_ESM.zip › EMBOJ-2024-119352_EVSourceData-2/Figure EV3/EV3A.tif]

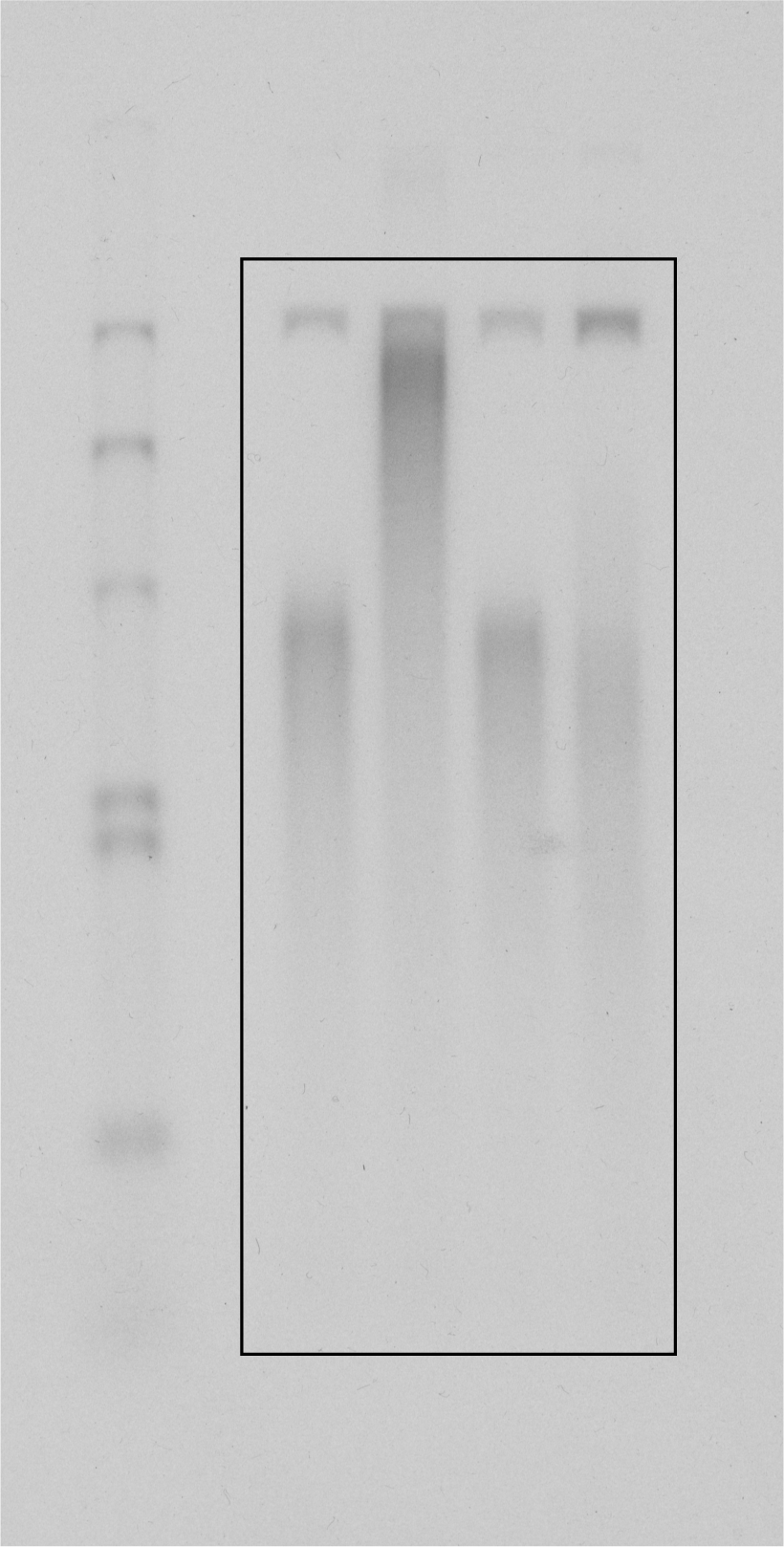

Supplement: Supplementary file 8 — EV Figures Source Data [file 44318_2025_386_MOESM8_ESM.zip › EMBOJ-2024-119352_EVSourceData-2/Figure EV2/EV2D.tif]

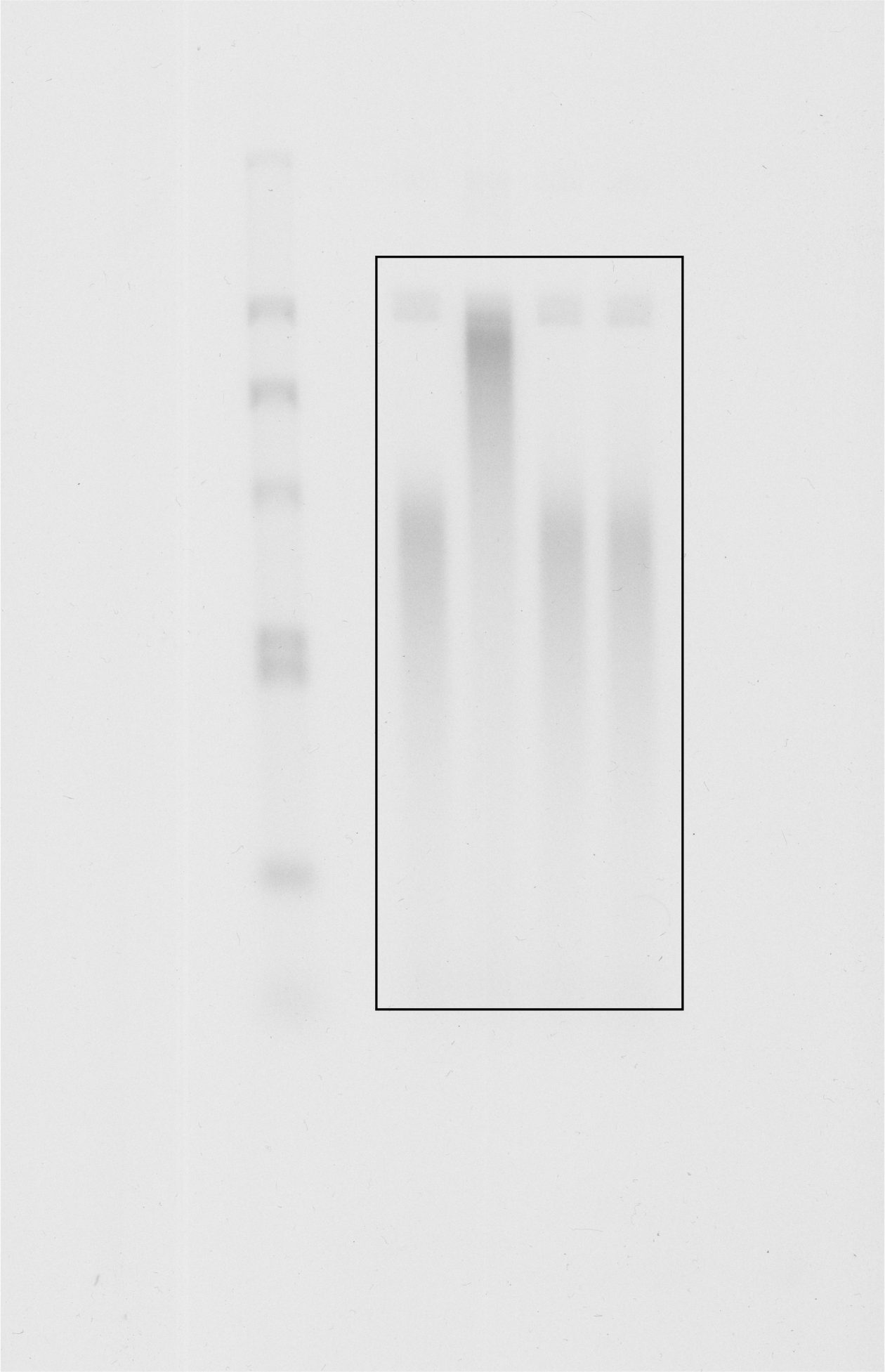

Supplement: Supplementary file 8 — EV Figures Source Data [file 44318_2025_386_MOESM8_ESM.zip › EMBOJ-2024-119352_EVSourceData-2/Figure EV2/EV2C.tif]

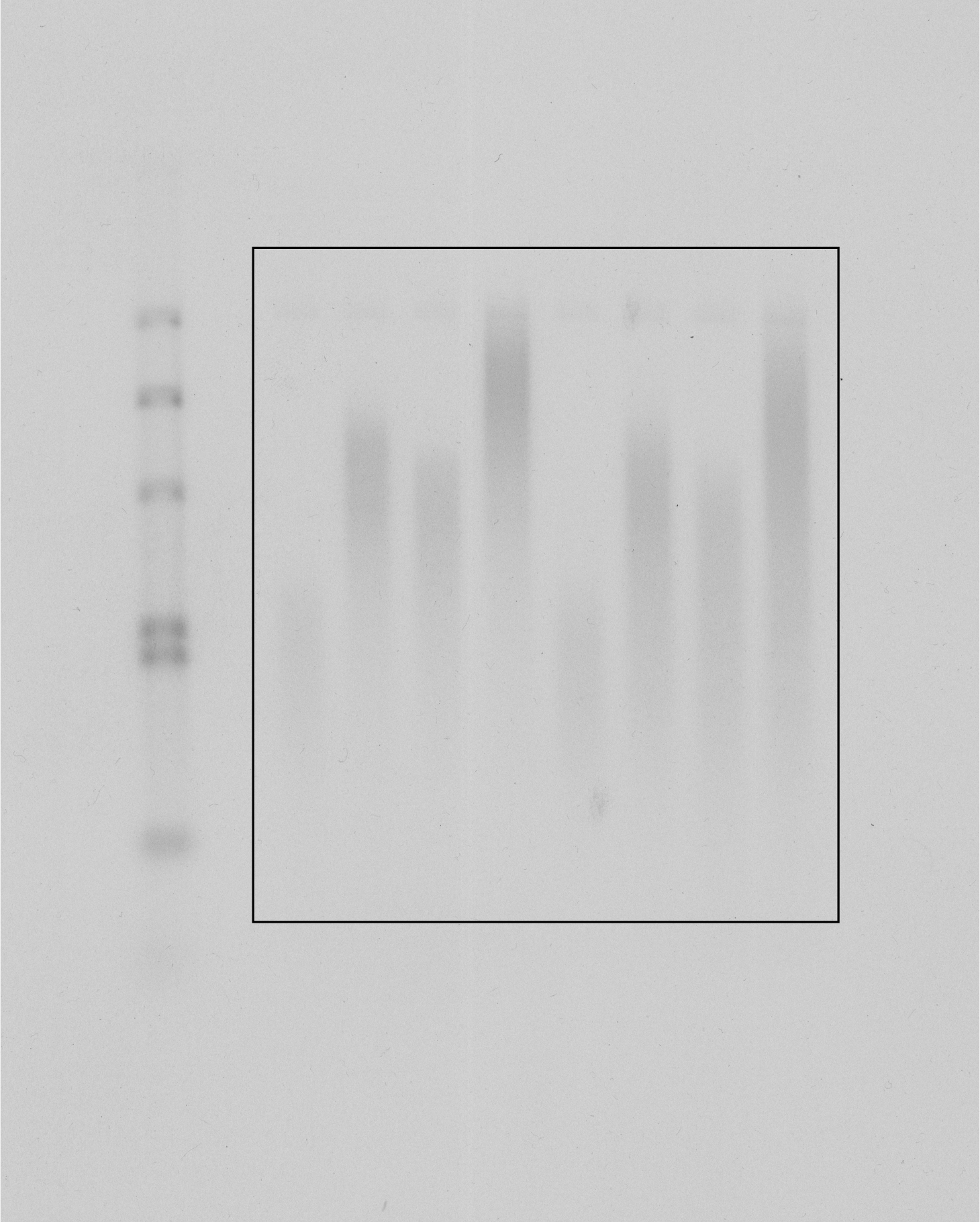

Supplement: Supplementary file 8 — EV Figures Source Data [file 44318_2025_386_MOESM8_ESM.zip › EMBOJ-2024-119352_EVSourceData-2/Figure EV6/EV6D.tif]

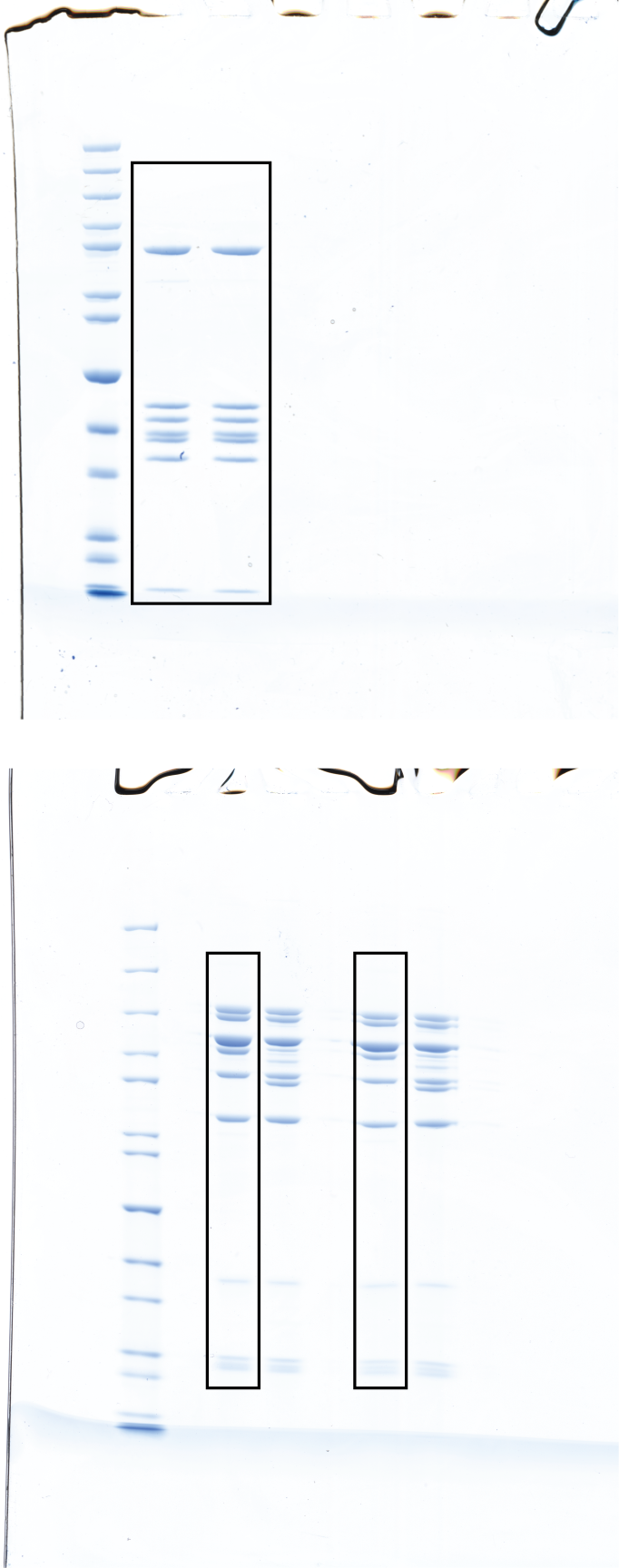

Supplement: Supplementary file 8 — EV Figures Source Data [file 44318_2025_386_MOESM8_ESM.zip › EMBOJ-2024-119352_EVSourceData-2/Figure EV6/EV6B.tif]

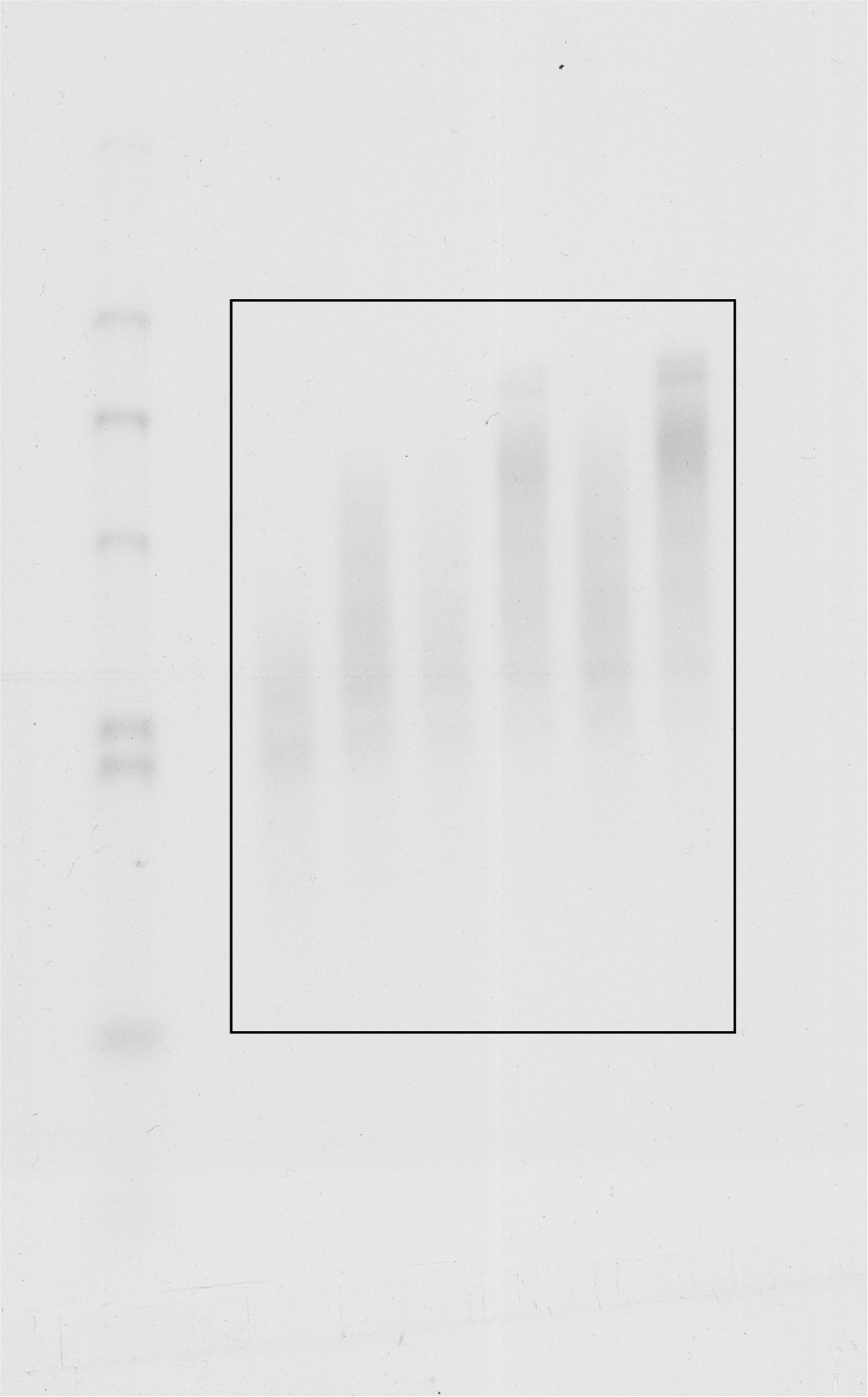

Supplement: Supplementary file 8 — EV Figures Source Data [file 44318_2025_386_MOESM8_ESM.zip › EMBOJ-2024-119352_EVSourceData-2/Figure EV6/EV6C.tif]

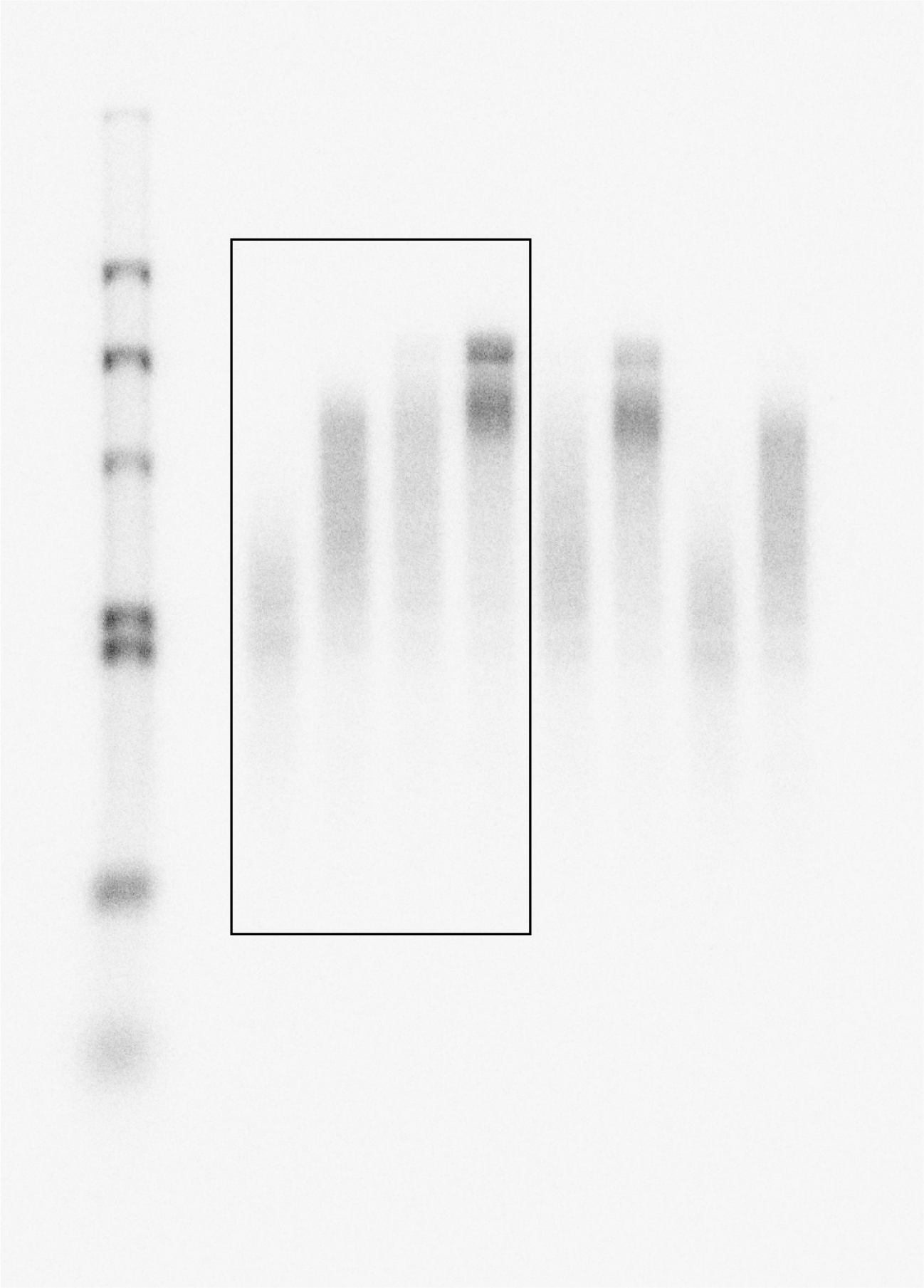

Supplement: Supplementary file 8 — EV Figures Source Data [file 44318_2025_386_MOESM8_ESM.zip › EMBOJ-2024-119352_EVSourceData-2/Figure EV1/EV1C.tif]

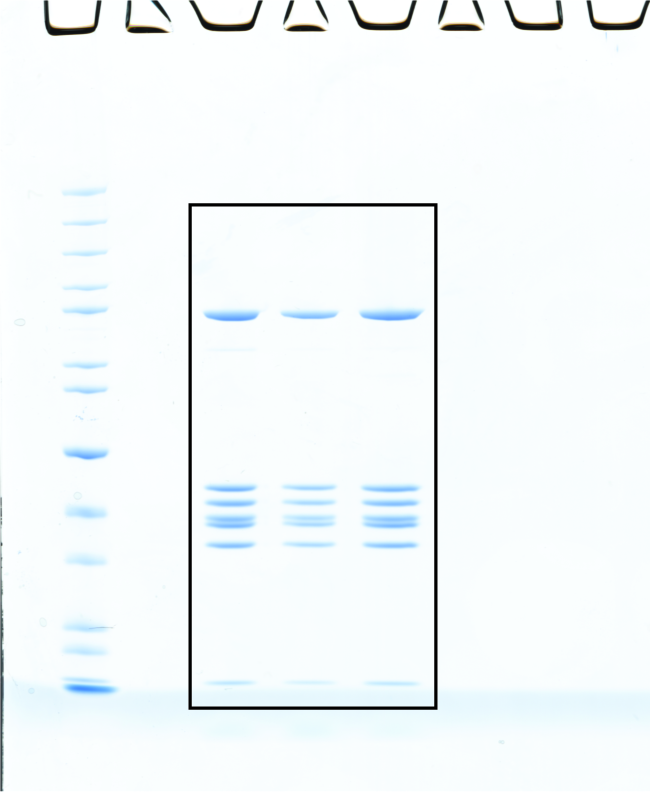

Supplement: Supplementary file 8 — EV Figures Source Data [file 44318_2025_386_MOESM8_ESM.zip › EMBOJ-2024-119352_EVSourceData-2/Figure EV1/EV1A.tif]

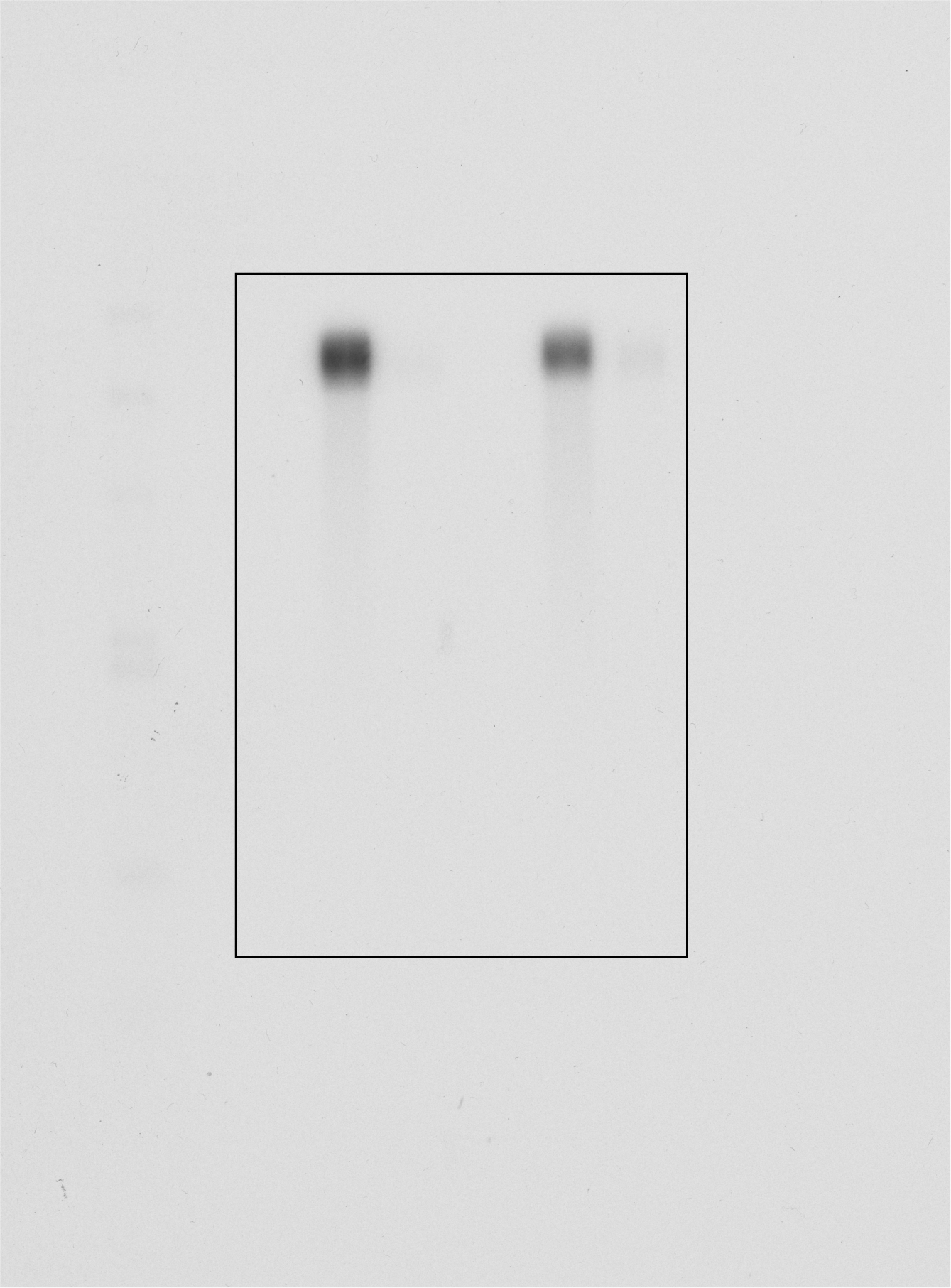

Supplement: Supplementary file 8 — EV Figures Source Data [file 44318_2025_386_MOESM8_ESM.zip › EMBOJ-2024-119352_EVSourceData-2/Figure EV1/EV1D.tif]
