# Supplementary material for: Competition for the nascent leading strand shapes the requirements for PCNA loading in the replisome
Source: EMBO J. 2025 Feb 28;44(8):2298–322. doi: 10.1038/s44318-025-00386-4 (PMC12000384; doi:10.1038/s44318-025-00386-4)
Supplement: Supplementary file 9 — Expanded View Figures [file 44318_2025_386_MOESM9_ESM.pdf]

Expanded View Figures

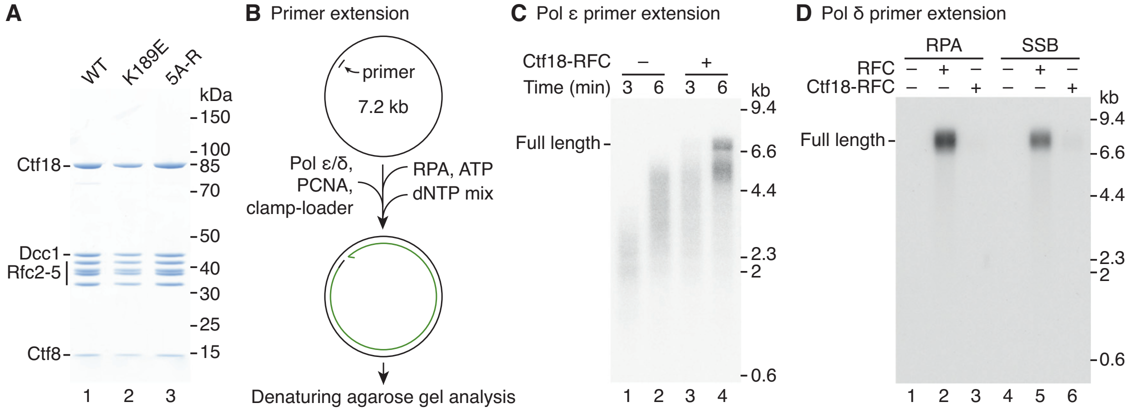

Figure EV1. Ctf18-RFC mutants and analysis of Ctf18-RFC in primer extension assays.

(**A**) Coomassie-stained 4–12% SDS-PAGE gel of purified *S. cerevisiae* wildtype (WT) or mutant Ctf18-RFC complexes. Subunits are labelled. (**B**) Schematic of a DNA polymerase primer extension reaction on circular ssDNA. (**C**) Denaturing agarose gel analysis of primer extension reactions as in (**B**) using Pol ε in the absence or presence of Ctf18-RFC. Reactions were performed at 100 mM potassium glutamate. (**D**) Denaturing agarose gel analysis of primer extension reactions as in (**B**) using Pol δ in the absence or presence of RFC or Ctf18-RFC, analysed after 1.5 min. The ssDNA template was coated with either *S. cerevisiae* RPA or *E. coli* SSB. Reactions were performed at 100 mM potassium glutamate. Source data are available online for this figure.

© The Author(s)

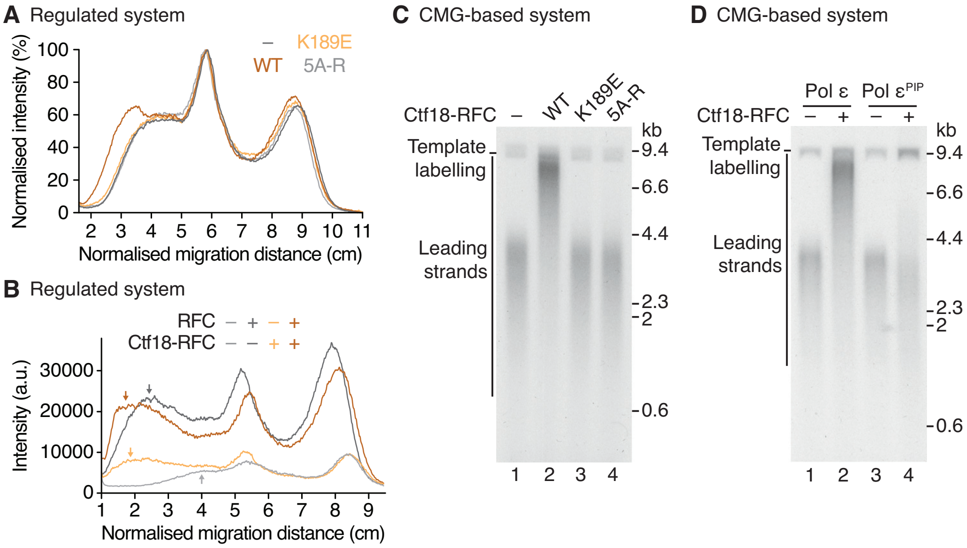

Figure EV2. (Related to Figs. 1 and 2). Analysis of leading-strand synthesis acceleration by Ctf18-RFC in the regulated and CMG-based systems.

(**A**) Lane profiles of 4 min timepoints from replication reactions using the regulated system, with wildtype (WT) or mutant Ctf18-RFC complexes present where indicated as in Fig. 1E. (**B**) Lane profiles of 5 min timepoints from replication reactions using the regulated system, in the absence and presence of RFC and Ctf18-RFC as in Fig. 1F. Arrows indicate leading-strand populations. (**C**) Denaturing agarose gel analysis of replication reactions using the CMG-based system, with wildtype (WT) or mutant Ctf18-RFC complexes present where indicated. Reactions were analysed after 3 min. (**D**) Denaturing agarose gel analysis of replication reactions using the CMG-based system with Pol ε or Pol ε^{PIP}, in the absence or presence of Ctf18-RFC. Reactions were analysed after 3 min. Source data are available online for this figure.

 © The Author(s)

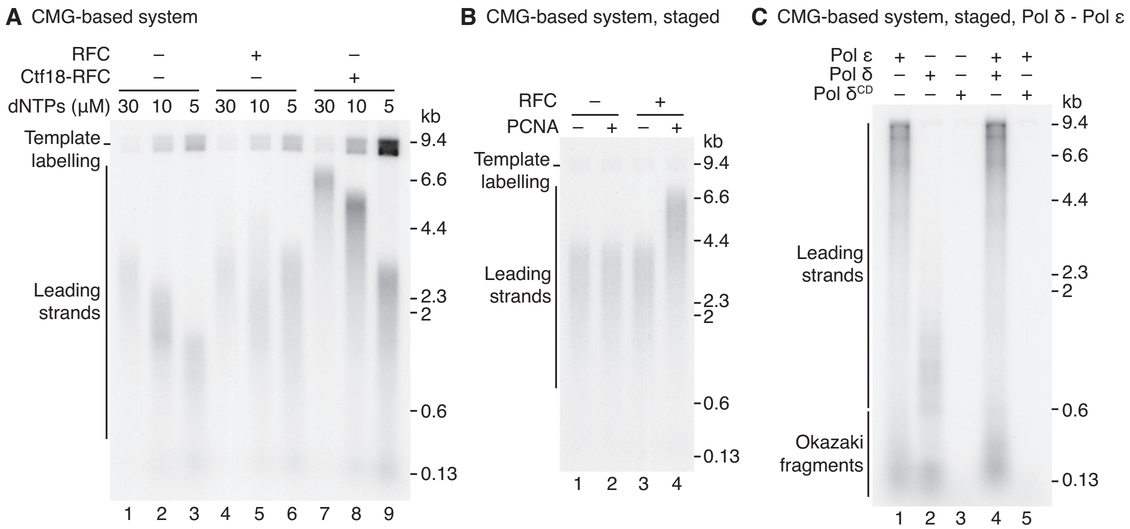

Figure EV3. (Related to Figs. 3 and 4). Analysis of reduced dNTP and staged CMG-based system assays.

(**A**) Denaturing agarose gel analysis of replication reactions using the CMG-based system in the absence or presence of RFC or Ctf18-RFC, with reduced dNTP concentrations as indicated. Reactions were analysed after 2.5 min. (**B**) Denaturing agarose gel analysis of replication reactions using the staged CMG-based system with Pol ε addition 1 min after initiation of template unwinding. PCNA and RFC were included where indicated. Reactions were analysed 4 min after initiation of template unwinding. (**C**) Denaturing agarose gel analysis of replication reactions using the staged CMG-based system but with Pol ε and Pol δ/ Pol $\delta^{CAT\text{-}DEAD}$ (Pol δ^{CD}) added 1 min after initiation of template unwinding where indicated. Pol α-primase was included with polymerase addition throughout. Reactions were analysed 5 min after initiation of template unwinding. Source data are available online for this figure.

© The Author(s)

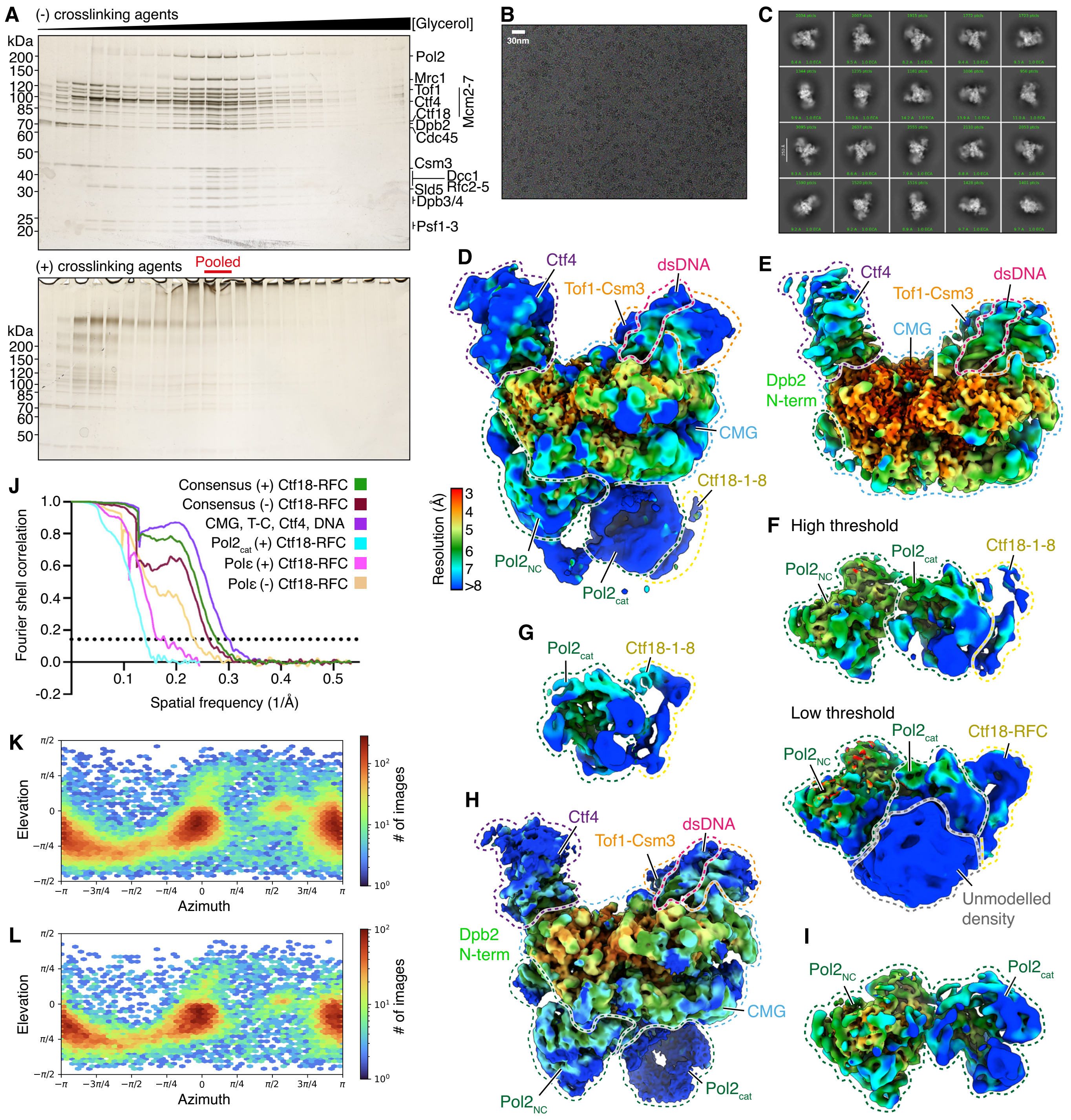
A
(-) crosslinking agents
[Glycerol]
kDa
200
150
120
100
85
70
60
50
40
30
25
20
Pol2
Mrc1
Tof1
Ctf4
Ctf18
Dpb2
Cdc45
Mcm2-7
Csm3
Dcc1
Rfc2-5
Sld5
Dpb3/4
Psf1-3
(+) crosslinking agents
Pooled
B
30nm
C
D
Ctf4
Tof1-Csm3
dsDNA
CMG
Ctf18-1-8
Pol2NC
Pol2cat
Resolution (Å)
3
4
5
6
7
>8
E
Ctf4
Tof1-Csm3
dsDNA
CMG
Dpb2
N-term
F
High threshold
Pol2NC
Pol2cat
Ctf18-1-8
Low threshold
Ctf18-RFC
Unmodelled
density
G
Pol2cat
Ctf18-1-8
H
Ctf4
Tof1-Csm3
dsDNA
Dpb2
N-term
CMG
Pol2NC
Pol2cat
I
Pol2NC
Pol2cat
J
Consensus (+) Ctf18-RFC
Consensus (-) Ctf18-RFC
CMG, T-C, Ctf4, DNA
Pol2cat (+) Ctf18-RFC
Polε (+) Ctf18-RFC
Polε (-) Ctf18-RFC
Fourier shell correlation
Spatial frequency (1/Å)
K
Elevation
Azimuth
# of images
L
Elevation
Azimuth
# of images

 © The Author(s)

Figure EV4. (Related to Fig. 5). Cryo-EM analysis of a budding yeast replisome prepared with Ctf18-RFC.

(**A**) Silver-stained SDS-PAGE gels analysing 100 µl fractions taken across 10-30% glycerol gradients, either in the absence (top) or presence (bottom) of crosslinking agents. Fractions 11-12 used for cryo-EM sample preparation are indicated with a red bar labelled "pooled". Protein annotations are based on the position of bands in lane 12. (**B**) Representative cryo-EM micrograph obtained using a K3 direct electron detector (Gatan) at a nominal pixel size of 0.93 Å/pixel. Scale bar: 30 nm (inset). (**C**) Representative 2D class averages with corresponding particle numbers. Derived from 47,823 particle subset used to obtain EMD maps 52107, 52116, 52120. Mask diameter 500 Å. Obtained using cryoSPARC-3 2D classification. (**D–I**) Cryo-EM reconstructions obtained using homogeneous or local refinement, coloured according to local resolution according to the key in (**D**). Local resolution estimation and filtering was performed in cryoSPARC-3. (**D**) Replisome with Ctf18-RFC bound to the Pol ε catalytic domain. The 47,823 particle subset used for this homogenous refinement was also used to derive maps EMD-52107, EMD-52116 and EMD-52120. (**E**) Local refinement following particle subtraction showing CMG, Tof1-Csm3, dsDNA, Ctf4 and $Dpb2_{N\text{-}term}$. EMD-52107, derived from particle subset in (**D**). (**F**) Local refinement following particle subtraction, showing Pol ε, Ctf18-RFC and Mcm5 WH. EMD-52116, derived from particle subset in (**D**). (Top) map at high threshold, (bottom) same map at low threshold highlighting presence of unmodelled density. (**G**) Local refinement following particle subtraction, showing the Pol ε catalytic domain and Ctf18-RFC. EMD-52120, derived from particle subset in (**D**). (**H**) Replisome lacking Ctf18-RFC bound to the Pol ε catalytic domain. EMD-52459, obtained from a homogenous refinement of a 20,462 particle subset. (**I**) Local refinement following particle subtraction, showing Pol ε and Mcm5 WH. EMD-52505, derived from particle subset in (**H**). (**J**) Fourier shell correlation (FSC) graph describing the maps in (**D–I**). Resolution was calculated using the FSC = 0.143 cut-off with values reported in Appendix Figs. S1–S3. (**K**, **L**) Viewing direction plots. 2D-histograms that show the number of particles with a viewing direction at a particular elevation/azimuth bin. (**K**) Consensus refinement of the replisome with Ctf18-RFC bound, as in (**D**). (**L**) Consensus refinement of the replisome without Ctf18-RFC bound, as in (**H**). Source data are available online for this figure.

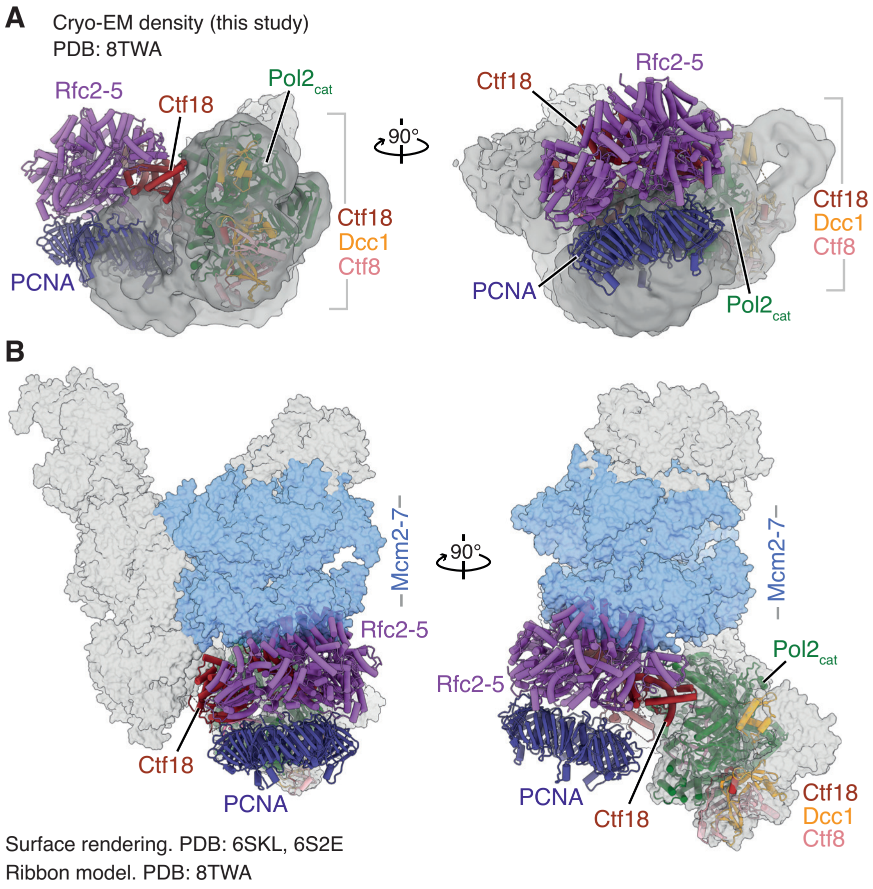

Figure EV5. (Related to Fig. 5). Structural comparisons of the replisome bound to Ctf18-RFC (this study) with relevant previously published structures.

(**A**) Model for Pol ε catalytic domain–DNA–Ctf18-RFC–PCNA complex (PDB:8TWA (Yuan et al, 2024)) docked into the cryo-EM density for Pol ε–Ctf18-RFC obtained in this study (EMD-52116). The Pol ε catalytic domain from 8TWA was rigid body docked into EMD-52116 using the "fit-in-map" command in ChimeraX. Docking highlights how the Ctf18-RFC ATPase module in 8TWA adopts an alternative position relative to Pol ε compared to that observed in this study. (**B**) Model for Pol ε catalytic domain–DNA–Ctf18-RFC–PCNA complex (PDB:8TWA) aligned to a model for the budding yeast replisome, where the Pol ε catalytic domain is positioned below the Mcm2-7 C-tier. To generate this replisome model, the previously published structure of the budding yeast replisome (PDB:6SKL (Baretic et al, 2020)) was rigid body docked into EMD-52107, and the Pol ε catalytic domain bound to Ctf18-RFC (PDB:6S2E (Stokes et al, 2020)) was docked into EMD-52116. The Pol ε catalytic domain of 8TWA was then aligned using Matchmaker in ChimeraX to the Pol ε catalytic domain of 6S2E. This alignment reveals how the conformation of the Ctf18-RFC ATPase module in 8TWA clashes with the C-tier of the Mcm2-7 helicase when the replisome adopts this configuration.

 © The Author(s)

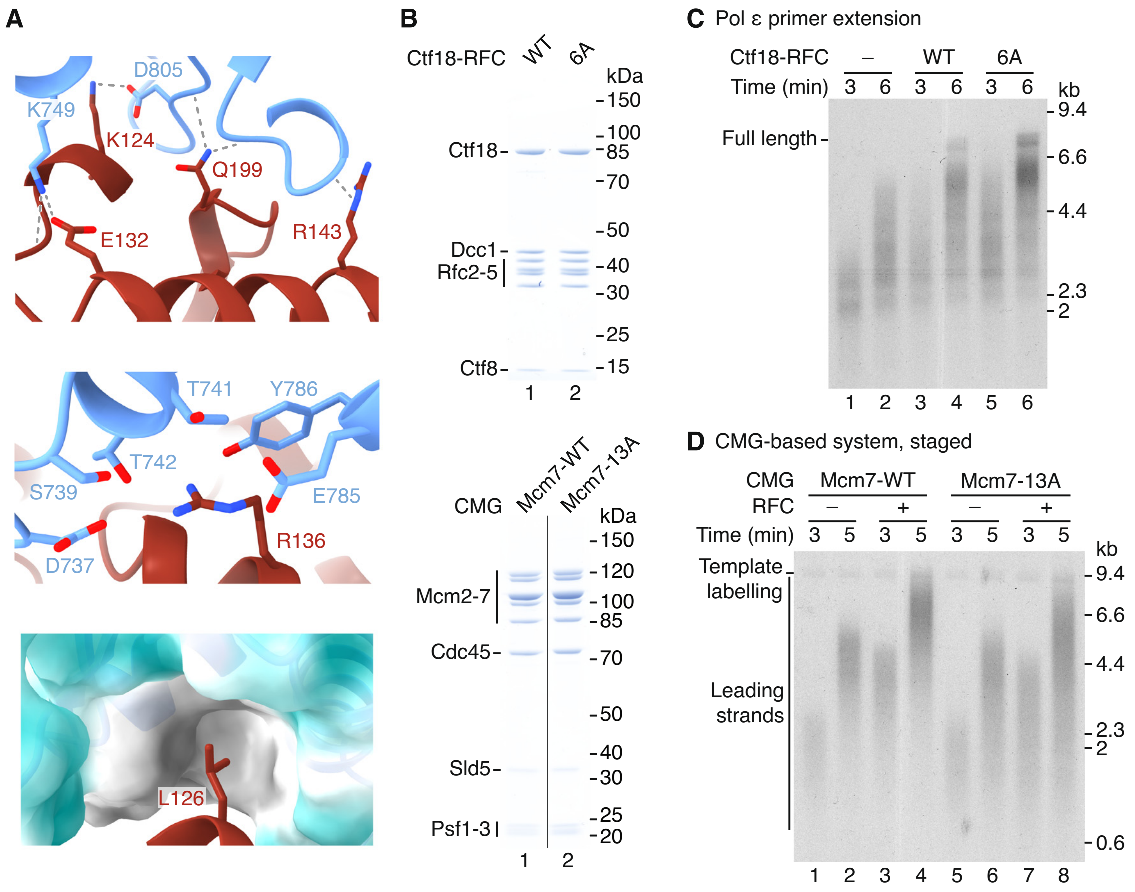

Figure EV6. (Related to Fig. 6). Analysis of mutants designed to disrupt the interface between Ctf18 and the Mcm7 WH.

(**A**) Detailed views of the AlphaFold predicted interaction between *S. cerevisiae* Ctf18 (red) and Mcm7 730–845 (blue). In the third panel, the Mcm7 WH molecular surface is rendered by hydrophobicity according to the Kyte-Doolittle scale, with hydrophilic regions in cyan and hydrophobic regions in grey. (**B**) Coomassie-stained 4–12% SDS-PAGE gel of purified *S. cerevisiae* wildtype (WT) or mutant Ctf18-RFC and CMG complexes. Subunits are labelled. (**C**) Denaturing agarose gel analysis of a Pol ε primer extension reaction with wildtype (WT) or mutant Ctf18-RFC complexes present where indicated. Reactions were performed at 100 mM potassium glutamate. (**D**) Denaturing agarose gel analysis of replication reactions using the staged CMG-based system with Pol ε addition 1 min after initiation of template unwinding. Wildtype (WT) or mutant CMG complexes and RFC were present where indicated. Source data are available online for this figure.

© The Author(s)
